# Pan-Cancer Analysis Reveals the Functional Importance of Protein Lysine Modification in Cancer Development

**DOI:** 10.3389/fgene.2018.00254

**Published:** 2018-07-17

**Authors:** Li Chen, Yanyan Miao, Mengni Liu, Yanru Zeng, Zijun Gao, Di Peng, Bosu Hu, Xu Li, Yueyuan Zheng, Yu Xue, Zhixiang Zuo, Yubin Xie, Jian Ren

**Affiliations:** ^1^State Key Laboratory of Oncology in South China, Cancer Center, Collaborative Innovation Center for Cancer Medicine, School of Life Sciences, Sun Yat-sen University, Guangzhou, China; ^2^Spine Center, Department of Orthopaedics, Anhui Provincial Hospital, The First Affiliated Hospital of USTC, Hefei, China; ^3^Department of Biomedical Engineering, College of Life Science and Technology, Huazhong University of Science and Technology, Wuhan, China

**Keywords:** lysine modifications, cancer, somatic mutations, clinical analysis, pathway and network analysis

## Abstract

Large-scale tumor genome sequencing projects have revealed a complex landscape of genomic mutations in multiple cancer types. A major goal of these projects is to characterize somatic mutations and discover cancer drivers, thereby providing important clues to uncover diagnostic or therapeutic targets for clinical treatment. However, distinguishing only a few somatic mutations from the majority of passenger mutations is still a major challenge facing the biological community. Fortunately, combining other functional features with mutations to predict cancer driver genes is an effective approach to solve the above problem. Protein lysine modifications are an important functional feature that regulates the development of cancer. Therefore, in this work, we have systematically analyzed somatic mutations on seven protein lysine modifications and identified several important drivers that are responsible for tumorigenesis. From published literature, we first collected more than 100,000 lysine modification sites for analysis. Another 1 million non-synonymous single nucleotide variants (SNVs) were then downloaded from TCGA and mapped to our collected lysine modification sites. To identify driver proteins that significantly altered lysine modifications, we further developed a hierarchical Bayesian model and applied the Markov Chain Monte Carlo (MCMC) method for testing. Strikingly, the coding sequences of 473 proteins were found to carry a higher mutation rate in lysine modification sites compared to other background regions. Hypergeometric tests also revealed that these gene products were enriched in known cancer drivers. Functional analysis suggested that mutations within the lysine modification regions possessed higher evolutionary conservation and deleteriousness. Furthermore, pathway enrichment showed that mutations on lysine modification sites mainly affected cancer related processes, such as cell cycle and RNA transport. Moreover, clinical studies also suggested that the driver proteins were significantly associated with patient survival, implying an opportunity to use lysine modifications as molecular markers in cancer diagnosis or treatment. By searching within protein-protein interaction networks using a random walk with restart (RWR) algorithm, we further identified a series of potential treatment agents and therapeutic targets for cancer related to lysine modifications. Collectively, this study reveals the functional importance of lysine modifications in cancer development and may benefit the discovery of novel mechanisms for cancer treatment.

## Introduction

Somatic mutations have a crucial role in the regulation of cancer progression, and therefore, interpreting the functional consequences of somatic mutations on gene products will be essential for developing potential targets for cancer therapies. As a benefit of the recent advances in next-generation sequencing technology and reduced analysis costs, the amount of data regarding somatic mutations in various cancer types has increased enormously in the past few years. A complex landscape of somatic mutations in cancers of multiple types and tissues has been revealed in large-scale cancer genomic datasets, such as TCGA and The International Cancer Genome Consortium (ICGC). However, among these massive amounts of mutations, not all of them are real drivers for cancer; instead, the majority of the mutations do not have a noticeable effect. Therefore, distinguishing only a few driver mutations from the majority of passenger mutations present in a cohort of patients is still a key challenge in the analysis of cancer genomes.

According to previous studies (Diaz-Cano, 2012; Morris et al., 2016), there is a significant genetic heterogeneity within the driver mutations presented in various cancer types. One possible explanation for this phenomenon is that the behavior of a cancer cell depends not only on genetic mutations but also on the dynamic regulation of non-genetic information. Therefore, combining mutations with other non-genetic regulations is an effective approach for predicting novel cancer driver genes and may provide extra guidance for cancer studies compared to traditional frequency-based methods (Vandin et al., 2012; Chen et al., 2013; Gonzalez-Perez et al., 2013; Leiserson et al., 2013; Tamborero et al., 2013; Cheng et al., 2014; Zhang et al., 2014). Protein post-translational modifications (PTMs), which are known to play critical roles in cancer development (Bode and Dong, 2004; Krueger and Srivastava, 2006; Jin and Zangar, 2009; Silvera et al., 2010), are an important functional feature that can be used in the prediction of novel cancer drivers. Among various protein amino acid residues, lysine has comparatively extensive and important modifications, such as acetylation, methylation and ubiquitination (Freiman and Tjian, 2003). The modification of lysine by ubiquitin and SUMO on specific proteins can reshape the binding interface between the modified protein and other biological macromolecules, regulating their affinity for DNA, proteins, plasma membranes or other endomembrane systems (Bergink and Jentsch, 2009; Dikic et al., 2009). Therefore, mutations of these modification sites may cause malfunction in PTM process, altering the subcellular location or activity of the modified protein and leading to abnormal functionalities (Ea et al., 2006). Recent research has reported that aberrant levels of histone acetylation can promote oncogenic transformation and tumorigenesis by deregulating chromatin-based processes (Lee et al., 2014; Di Martile et al., 2016). As research moves forward, growing evidence has shown that the acetylation process on non-histone proteins (Glozak et al., 2005; Singh et al., 2010), especially transcription factors, is highly related to cancer phenotype. In addition to lysine acetylation, SUMOylation, and ubiquitination were also found to be involve in cancer progression. For example, the mutation of E318K on melanoma-lineage-specific microphthalmia-associated transcription factor (MITF) can disrupt a SUMO consensus site, and lack of SUMOylation increased the transcriptional activity of MITF, thereby increasing the levels of other tumor promoting factors, such as HIF1α (Bertolotto et al., 2011; Yokoyama et al., 2011). Thus, aberrant SUMOylation of MITF promotes tumor initiation and progression. In addition to MITF, another key cancer driver that is regulated by lysine modification is the androgen receptor (AR). According to previous evidence (Heinlein and Chang, 2004; Balk and Knudsen, 2008; Tan et al., 2015), AR is the main driver of prostate cancer development and progression. Recent research has revealed that the ubiquitination of AR at position K311 is critical for its proper function, regulating both AR protein stability and AR transcriptional activity. When such an ubiquitination site loses its function, the expression of over a thousand downstream genes will be altered, possibly leading to misregulation in chromatin organization, cellular adhesion, motility, and signal transduction (McClurg et al., 2016). In this regard, the annotation of known cancer mutations based on the effects on lysine modification and the discovery of novel lysine modification-related drivers may be important for providing potential guidance in the development of new therapeutic strategies and drugs for cancer patients.

To uncover the potential mechanism of lysine modification in cancer development, here, we collected 1,085,623 somatic mutations and 103,248 lysine modification sites from existing databases and published literatures. Combining the above data together, we identified 164,884 lysine modification-related mutations. To further predict driver proteins that carried recurrent mutations on lysine modification sites, we developed a hierarchical Bayesian model and applied a Markov Chain Monte Carlo (MCMC) method for analysis. Furthermore, based on the identified driver proteins, we performed comprehensive downstream analysis to reveal their regulatory roles in biological pathways and molecular interaction networks. In addition, their potential clinical utilities in cancer diagnosis and treatment were also evaluated in this study. We expect that the above information will help in the discovery of novel mutagenic mechanisms and therapeutic targets for cancer studies.

## Materials and methods

### Collection of lysine modification sites and non-synonymous mutations

Experimentally identified lysine modification sites in human proteins and their exact sequences were manually collected from published literatures in PubMed by searching for keywords such as “ubiquitination,” “acetylation,” “sumoylation,” “methylation,” “succinylation,” “malonylation,” “glutarylation,” “glycation,” “formylation,” “hydroxylation,” “butyrylation,” “propionylation,” “crotonylation,” “pupylation,” “neddylation,” “2-hydroxyisobutyrylation,”“phosphoglycerylation,” “carboxylation,” “lipoylation,” and “biotinylation.” To ensure an adequate amount of data, only the modifications with more than 1,000 sites were retained for subsequent analysis. Ultimately, seven types of lysine modifications (ubiquitination, acetylation, SUMOylation, glycation, malonylation, methylation, and succinylation) were collected as the final data set. All modified proteins were annotated with Ensembl transcripts and HGNC symbols using the UniProt database.

Somatic mutations of 12 cancer types (BLCA, UCEC, LAML, STAD, SKCM, GBM, THCA, LIHC, HNSC, COAD, LUSC, and THYM) were downloaded from the TCGA data portal (https://portal.gdc.cancer.gov/) on 16 March 2017. To obtain a complete set of mutation data, we also downloaded somatic mutations of these cancer types from the ICGC data portal (ICGC, https://dcc.icgc.org/, downloaded on 21 November 2017) and the Catalog of Somatic Mutations in Cancer (COSMIC Forbes et al., 2017, downloaded on 21 November 2017). The original mutation sites were then combined from the above three databases, and redundant mutations in the same patients in the same cancer type were removed. After annotation by ANNOVAR (Wang et al., 2010), only non-synonymous single nucleotide variants (SNVs) were retained.

### Identification of lysine modification-related mutations from mutation data

We applied a k-means clustering algorithm to extract the corresponding motif regions of lysine modifications (Supplementary Methods, Supplementary Table 1). We merged the motifs of the same type of lysine modification in order at integrated PTM sites with cancer mutations for each protein and then denoted them as the modification regions. Correspondingly, the remaining sequences were merged separately and denoted as background regions (**Figure 2**). Non-synonymous mutations were mapped to the protein sequences and divided into lysine modification-related mutations, which were located in the modification region, and modification-irrelevant mutations, which were located in the background region. We only focused on proteins with at least one lysine modification and discarded other non-modified proteins to avoid systematic biases.

### Analysis of lysine modification-related driver proteins by hierarchical Bayesian model

To identify proteins with significantly altered numbers of lysine modification-related mutations, we then constructed the following hierarchical Bayesian model. In our model, we first assumed that mutations on the motif regions would probably damage the lysine modification process, thereby influencing the function of their corresponding proteins via PTM-related pathways. If such mutations are highly correlated with tumor proliferation, they will probably undergo strong positive selection during the cancer development process, and therefore, unexpectedly high mutation rates will be observed in these regions. In view of this assumption, we can identify lysine modification-related driver proteins by comparing the mutation rates in both motif regions and modification-free regions. Accordingly, a null hypothesis that the mutation rate in the motif region is same as the mutation rate in the modification-free region is proposed. More formally, we describe the detailed computational process below.

First, for a given protein, let *Y*_1_, *Y*_2_, ⋯*Y*_*k*_ represent the number of somatic mutations in each position in the modification region, and *Y*_*k*+1_, *Y*_*k*+2_, ⋯*Y*_*n*_ be the same count in the background region. According to this definition, the observed counts *Y* can be described by a Poisson distribution as shown in Equations (1) and (2), where λ_1_ and λ_2_ are the mutation rates of the modification region and the background region, respectively.

(1) $$\begin{array}{l} Y_{i}\sim \;Poisson\left(\lambda_{1}\right)\begin{array}{c} i=1,2,\cdots \;,k \end{array} \end{array}$$

(2) $$\begin{array}{l} Y_{i}\sim \;Poisson\left(\lambda_{2}\right)\begin{array}{c} i=k+1,k+2,\cdots \;,n \end{array} \end{array}$$

However, due to heterogeneity in the mutational spectrum of tumors, the mutation rate may vary markedly within different regions across different cancer types (Lawrence et al., 2013). To capture this fluctuation, a prior distribution was applied on λ_1_ and λ_2_ to build a double hierarchical model. As stated in the theory of probability, a gamma distribution is the conjugate prior to the Poisson distribution. Therefore, two gamma distributions with different shape parameters α and scale parameters β were used to describe the distribution of λ_1_ and λ_2_ in Equations (3) and (4):

(3) $$\begin{array}{l} \lambda_{1}\sim \;Gamma\left(\alpha_{1},\beta_{1}\right) \end{array}$$

(4) $$\begin{array}{l} \lambda_{2}\sim \;Gamma\left(\alpha_{2},\beta_{2}\right) \end{array}$$

To compare the mutation rates in the modification and background regions, we first need to compute the marginal distribution of λ_1_ and λ_2_ given the observed data *Y* in our hierarchical model, i.e., calculating *P*(λ_1_|*Y*) and *P*(λ_2_|*Y*). Therefore, a concrete from of the full joint probability should be obtained. According to Bayesian theory, the full joint probability can be written as shown in Equation (5) (see Supplementary Methods for detail deviation process).

(5) $$\begin{array}{l} P\left(\lambda_{1},\lambda_{2}|Y\right)=P\left(Y_{1:k}|\lambda_{1}\right)P\left(Y_{k+1:n}|\lambda_{2}\right)P\left(\lambda_{1}\right)P\left(\lambda_{2}\right)\\ =\prod_{i=1}^{k}e^{-\lambda_{1}}\frac{{\lambda_{1}}^{Y_{i}}}{Y_{i}!}\prod_{i=k+1}^{n}e^{-\lambda_{2}}\frac{{\lambda_{2}}^{Y_{i}}}{Y_{i}!}\frac{{\beta_{1}}^{\alpha_{1}}{\lambda_{1}}^{\alpha_{1}-1}e^{-\beta_{1}\lambda_{1}}}{\Gamma \left(\alpha_{1}\right)}\\ \;\frac{{\beta_{2}}^{\alpha_{2}}{\lambda_{2}}^{\alpha_{2}-1}e^{-\beta_{2}\lambda_{2}}}{\Gamma \left(\alpha_{2}\right)} \end{array}$$

However, computing the marginal distribution from the above full joint probability required integrating over other unrelated variables in Equation (5), which was generally a formidable analytic problem and could hardly be done manually. Rather than mathematically computing the integration, estimating the marginal distributions by the MCMC method, i.e., Gibbs sampling, is a more straightforward approach. Therefore, in order to implement Gibbs sampling (Supplementary Figure 3), the full conditional posterior probability of every parameter should be calculated. As shown in the Supplementary Methods, the final full conditional posterior probability of λ_1_ and λ_2_ were obtained in

(6) $$\begin{array}{l} P\left(\lambda_{1}|\lambda_{2},Y\right)=Gamma\left(\overset{k}{\underset{i=1}{\sum }}Y_{i}+\alpha_{1},k+\beta_{1}\right) \end{array}$$

(7) $$\begin{array}{l} P\left(\lambda_{2}|\lambda_{1},Y\right)=Gamma\left(\overset{n}{\underset{i=k+1}{\sum }}Y_{i}+\alpha_{2},n-k+\beta_{2}\right) \end{array}$$

Equations (6) and (7).

To test the difference between the mutation rates of the background and modification regions, a variable of interest might be the relative mutation rate, which is defined as $R=\frac{\lambda_{1}}{\lambda_{2}}$. Similarly, the full conditional posterior probability can be calculated as shown in Equation (8) (Supplementary Methods).

(8) $$\begin{array}{l} P\left(R|\lambda_{1},\lambda_{2},Y\right)=Gamma\left(\sum_{i=1}^{k}Y_{i}+\alpha_{1},\lambda_{2}k+\lambda_{2}\beta_{1}\right) \end{array}$$

After calculating all full conditional probabilities of each variable, we can now use a Gibbs sampling algorithm to estimate the marginal distribution of these parameters. During the calculation, we performed 5,200 iterations in total and removed the first 200 iterations as a burn-in process. Finally, the marginal distribution of λ_1_, λ_2_ and *R* was estimated by the data sampled from the last 5,000 iterations. Given the null hypothesis raised at the very beginning of this section, we can rewrite the hypothesis as shown in Equation (9).

(9) $$\begin{array}{l} H_{0}:R\leq 1\\ H_{1}:R>1 \end{array}$$

The *p*-value under the null hypothesis is then calculated from the marginal distribution of *R*. For each tested protein, the probability of observing a relative mutation rate <1 can be calculated. To control false positives, the Benjamini-Hochberg procedure is applied to each *p*-value. If the corrected *p*-value for a given protein is lower than the significance level, i.e., 0.05, we identify it as a significantly mutated protein.

### Domain association analysis of lysine modification-related driver proteins

The functional domains of each candidate driver protein were first predicted from InterProScan (Jones et al., 2014) using the Pfam (Finn et al., 2014) and SMART databases (Schultz et al., 1998; Letunic et al., 2009). The predicted regions of each protein were then merged together to construct a domain region, and the remaining sequences were merged as a disorder region. To examine whether the lysine modification-related mutations occurred preferentially in the domain region than in the disorder region, we designed a two-tailed bootstrap tests to compare the number of lysine modification-related mutations in the domain and disorder region. The bootstrap test was performed according to the following steps.

First, for each protein, we used Equations (10) and (11) to calculate the number of mutations that occurred per thousand amino acids in the domain region and disorder region. Specifically, we denoted the above mutation number in the domain region and disorder region as *x* and *y*, respectively.

(10) $$\begin{array}{l} x=\frac{X}{l_{1}}\times 1000 \end{array}$$

(11) $$\begin{array}{l} y=\frac{Y}{l_{2}}\times 1000 \end{array}$$

where *X* and *Y* are the exact number of lysine modification-related mutations observed in the domain region and disorder region, respectively. *l*_1_ and *l*_2_ are the length of the domain region and disorder region, respectively.

Next, we tested the null hypothesis that *x* was equal to *y* in our observed data. To test this hypothesis, the probability of observing *x* not equal to *y* under the null distribution must be calculated. Therefore, we used the transformation in Equations (12) and (13) to estimate the null distribution. After the transformation in Equations (12) and (13), we can let the distribution of *x* and *y* be the same and constrain them to have the same center *z*.

(12) $$\begin{array}{l} {\tilde{x}}_{i}=x_{i}-\bar{x}+z \end{array}$$

(13) $$\begin{array}{l} {ỹ}_{j}=y_{j}-ȳ+z \end{array}$$

(14) $$\begin{array}{l} z=\frac{\sum_{i=1}^{m}x_{i}+\sum_{j=1}^{n}y_{j}}{m+n} \end{array}$$

In the above equation, *x*_*i*_ is the number of mutations located in the domain region for the *i*-th protein, whereas *y*_*j*_ is the number of mutations for disorder regions in the *j*-th protein. $\bar{x}$ and ȳ are the average number of mutations located in all domain regions and disorder regions, respectively. *m* and *n* represent the total number of mutations in the domain and disorder region, respectively.

Based on the above definition, we then constructed *B* bootstrap data sets (x^*^, y^*^) by sampling x^*^ with replacement from $\tilde{x}$ and y^*^ with replacement fromỹ. The test statistic $t_{b}^{*}$ was calculated as shown in Equation (15).

(15) $$\begin{array}{l} t_{b}^{*}=\frac{{\bar{x}}_{b}^{*}-{ȳ}_{b}^{*}}{\sqrt{\frac{{\hat{\sigma }}_{xb}^{*2}}{n}}+\sqrt{\frac{{\hat{\sigma }}_{yb}^{*2}}{m}}} \end{array}$$

where ${\bar{x}}_{b}^{*}$ is the mean and ${\hat{\sigma }}_{xb}^{*2}$ is the variance of the *b*th bootstrap sample $x_{b}^{*}$. The probability of observing *x* not equal to *y* under the null distribution can now be approximated by Equations (16) and (17).

(16) $$\begin{array}{l} p=\frac{\overset{\text{B}}{\underset{\text{b}=1}{\sum }}\text{I(}|{\text{t}}_{\text{b}}^{*}|\geq |{\text{t}}_{\text{obs}}|\text{)}}{\text{B}} \end{array}$$

(17) $$\begin{array}{l} I(x)=\{\begin{array}{cc} 1 & x=True\\ 0 & x=False \end{array} \end{array}$$

If the calculated *p*-value is lower than a pre-defined significance level, e.g., 0.05, then we should reject the null hypothesis and accept that the lysine modification mutations are more likely to be enriched in the domain region.

### Conservation and deleteriousness analysis of lysine modification-related mutations

The sequence conservation of each mutation site was quantified using the 100 way phastCons score calculated in ANNOVAR. The phastCons score was originally designed to identify conserved elements in multiply aligned sequences. Using a phylogenetic hidden Markov model (phylo-HMM), the probability of nucleotide substitutions occur at each site in a genome was quantitatively measured (Siepel et al., 2005). And based on such probability profile (i.e., phastCons score profile), one can calculate the conservation degree of a given mutation site. In this study, we used the phastCons scores to quantify the conservation degree of all the lysine modification-related mutations and other non-lysine modification mutations. Their cumulative distribution functions (CDF) were also plotted to present the differences.

To investigate that whether our identified lysine modification-related mutations was more probable to damage specific protein functions, we next introduced a deleterious scores in our study for measuring their deleteriousness. We defined that if a given SNV was found to disrupt the functional domains or regulation regions in a specific protein, such mutation would be deleterious to protein functions. Therefore, five pieces of software, including SIFT (Ng and Henikoff, 2001), PolyPhen2 HVAR, PolyPhen2 HDIV (Adzhubei et al., 2010), LRT (Chun and Fay, 2009), and FATHMM (Shihab et al., 2013), were adopted to predict the functional consequences of our identified lysine modification-related mutation sites. To ensure prediction accuracy, we further defined a deleterious score by integrating the prediction results from the above five software. Specifically, the deleterious score was calculated by counting the number of the above methods that considered a mutation to be deleterious. A deleterious score of 0 means that the mutation is predicted to be tolerated in all methods, whereas a deleterious score of 5 means that the corresponding mutation is predicted to be deleterious in all five predictors. As a result, the deleterious score may range from 0 to 5, and a higher score indicates a higher probability of deleterious. Next, a two-tailed proportion test was then applied to compare the deleterious difference between lysine modification-related mutations and other mutations.

### Subcellular location analysis

To annotate the subcellular location of our identified driver proteins, we first downloaded the data set from Thul's paper (Thul and Akesson, 2017). The identified driver proteins were then mapped to their corresponding subcellular locations according to the downloaded data set. Specifically, we categorized our driver proteins into 13 basic cellular compartments, which were the cytosol, mitochondria, microtubules, actin filaments, intermediate filaments, centrosome, nucleus, nucleoli, vesicles, plasma membrane, Golgi apparatus, ER, and secreted. The final annotation was summarized in a Venn diagram.

### Pathway enrichment analysis

To uncover the regulation roles of our identified driver proteins in cancers, we performed KEGG pathway and Gene Ontology enrichment analysis using the “clusterProfiler” (Yu et al., 2012) and “ReactomePA” (Yu and He, 2016) package in R. The analysis results were illustrated using bubble plots or Cytoscape (Demchak et al., 2014).

### Survival analysis

We downloaded survival data from the TCGA data portal (https://tcga-data.nci.nih.gov/docs/publications/tcga/?) and employed the R package “survival”(https://CRAN.R-project.org/package=survival) to obtain the distribution of overall survival time using Kaplan-Meier estimation. A log-rank test was used to compare the survival distributions of two groups: patients with mutations exactly located in PTM modification regions and patients with other mutations. Survival curves were plotted by the R package “survminer” (http://www.sthda.com/english/rpkgs/survminer).

## Results

### Global analysis reveals recurrent cancer mutations in lysine modification sites

**Figure 1:**
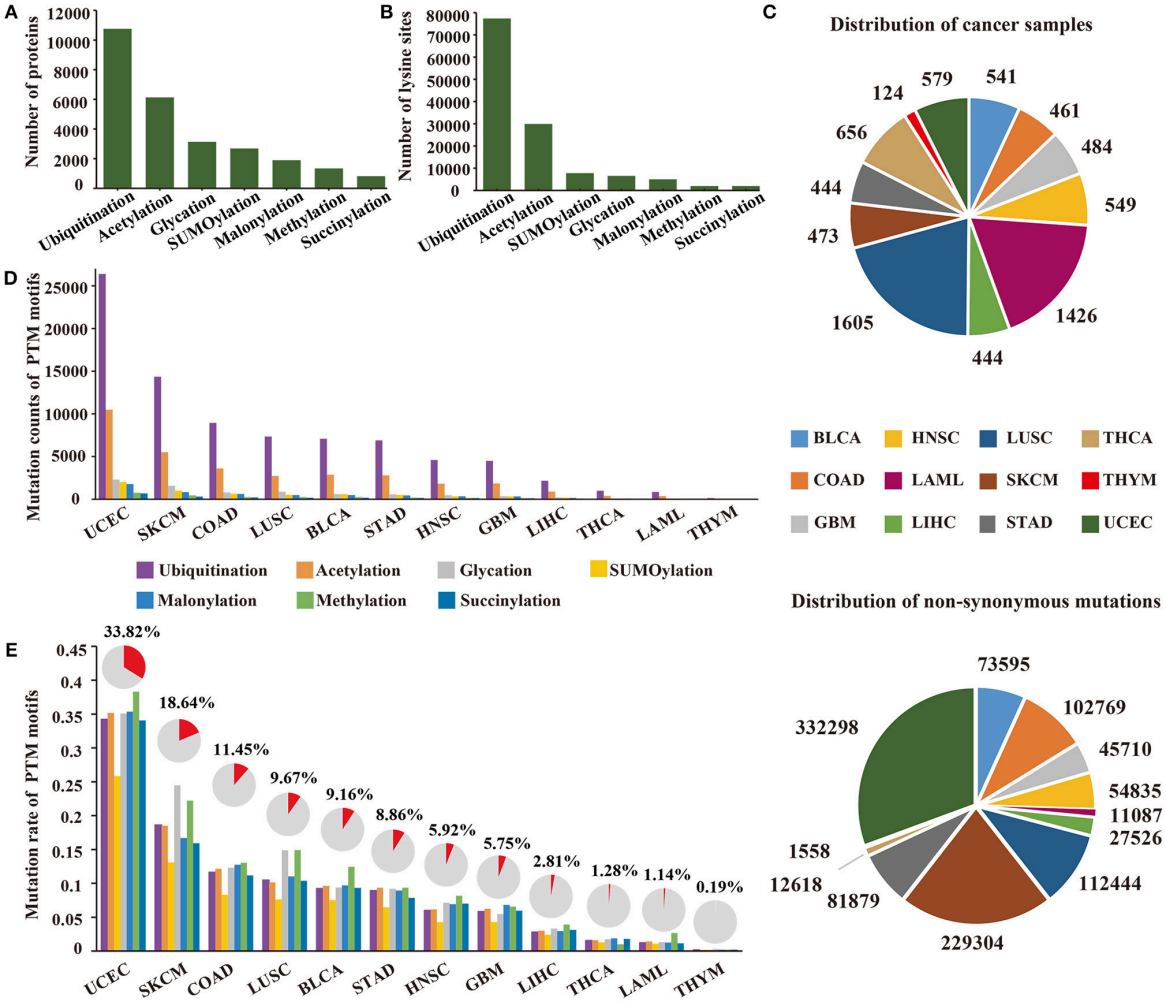
**(A)** The number of proteins with lysine modifications collected from published literatures. **(B)** The number of lysine modification sites collected in this study. **(C)** Distribution of cancer samples and somatic mutations collected across 12 cancer types. **(D)** The count of mutated PTM sites across 12 cancer types. **(E)** The count of mutated PTM motifs across 12 cancer types. The proportion of mutated lysine modification motifs are shown above the bar plot.

To investigate specific cancer mutations in lysine modifications, we collected 103,248 experimentally identified lysine modification sites in 13,378 proteins in total from published literatures (Figure 1A). The collected modification sites consisted of 77,364 ubiquitination sites, 29,942 acetylation sites, 7,821 SUMOylation sites, 6,568 glycation sites, 5,013 malonylation sites, 2,018 methylation sites, and 2,014 succinylation sites (Figure 1B, Supplementary Table 2). Considering the fact that modifications of lysine residues were mainly catalyzed by specific enzymes and that each enzyme has a quite different recognition motif, we first applied a modified k-means clustering algorithm to divide the modification sites into different consensus groups (Supplementary Figure 1). To determine the optimal recognition motif for each consensus group, we then carried out a PSSM-based method on the grouped data and visualized the amino acid preference with the Seq2Logo software (Thomsen and Nielsen, 2012). According to the calculated amino acid profiles (Supplementary Figure 2), we empirically selected the optimal length of the recognition motif and constructed the motif region for each consensus group.

Recent publications have revealed that amino acid mutations within the modification motif can disrupt the interaction between modification enzymes and specific amino acid residues, thereby altering the level of post-translational modification on specific proteins (Taverna et al., 2007; Reimand and Bader, 2013; Hornbeck et al., 2015). Therefore, a total of 1,085,623 non-synonymous mutations (Figure 1C) from 7,786 patients (Figure 1C) were collected from TCGA for subsequent analysis. By mapping the non-synonymous mutations to the motif regions, we finally obtained 164,884 lysine modification-related mutations from 12 selected cancers in 7 modification types (ubiquitination, acetylation, SUMOylation, glycation, malonylation, methylation, and succinylation) (Supplementary Table 3), which amounted to 68,401 damaged lysine modification sites (Figure 1D). Surprisingly, of the 12 selected cancer types, we observed that uterine corpus endometrial carcinoma carried the largest number of lysine modification-related mutations in its samples, and more than 33.8% of the modification sites were mutated in this cancer type (Figure 1E). These results demonstrated that abnormal lysine modification is a general mechanism of cancer cell regulation, implying its functional importance in different cancer types.

### Driver proteins with significant lysine modification-related mutations

**Figure 2:**
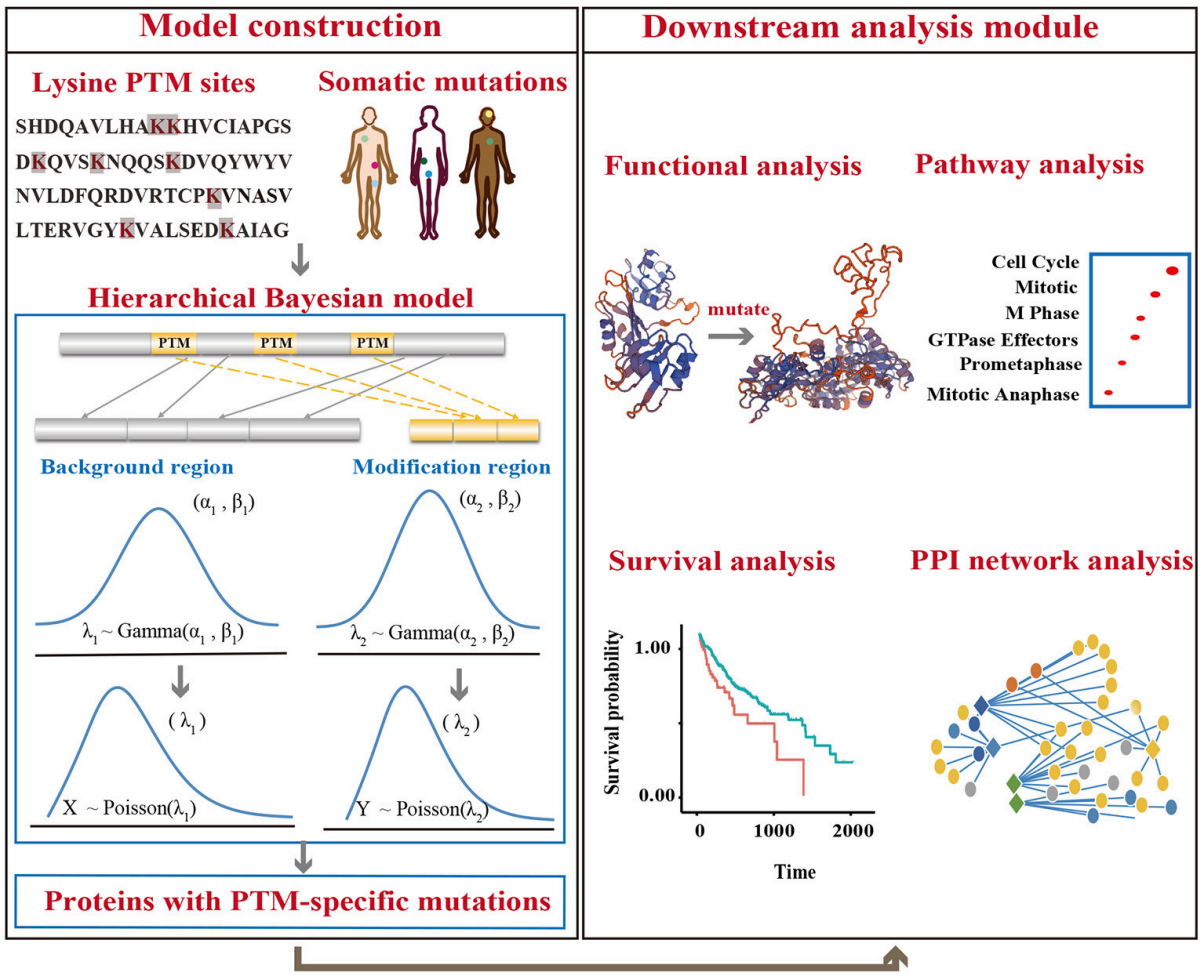
An overview of the analysis model. Lysine modification sites were collected from published literatures. Somatic mutations were downloaded from TCGA, ICGC, and COSMIC. A hierarchical Bayesian model was then constructed to identify proteins with mutations that were significantly enriched in PTM regions. Downstream analysis was also performed to reveal the mechanism of lysine modification-related mutations in cancers.

To identify driver proteins carrying significant lysine modification-related mutations in multiple cancer types, we developed a hierarchical Bayesian model and applied the MCMC method to estimate the mutation frequency in modification regions (Figure 2, Supplementary Methods). We assumed that for a given protein, if the mutation frequency observed in the motif region was higher than that the non-motif region, the modification process in this protein may undergo obvious positive selection and the corresponding mutations may have a significant effect on protein function. Therefore, identifying proteins that carried a higher mutation rate in the lysine modification site could assist with finding targets that potentially drive cancer progression. In this regard, a null hypothesis that the mutation rate in motif regions is equal to that in non-motif regions was proposed in our Bayesian model. For each tested protein, the *p*-value of observing a higher mutation rate in motif regions than in non-motif regions was calculated. In addition, the Benjamini-Hochberg method was then applied to control the false discover rate in the statistical test.

**Figure 3:**
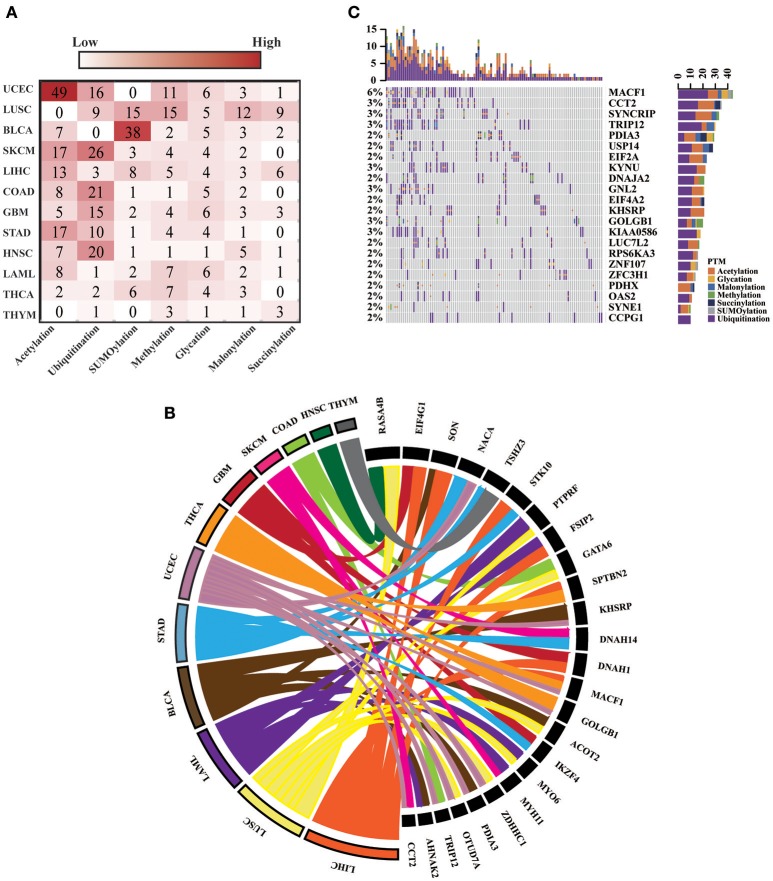
**(A)** The heatmap shows the number of significantly mutated lysine modification-related proteins across 7 modification types in 12 cancers. **(B)** The 25 driver proteins that mutated in more than one cancer type are shown in the Circos plot. The width of the lines that connect mutated proteins to cancer types denotes the log_10_ value of the fold change between modification regions and background regions. Different colors represent different cancer types. **(C)** Oncoprint for lysine modification-related mutations in UCEC. The number of mutations in each patient or protein are visualized in the bar graph.

**Figure 4:**
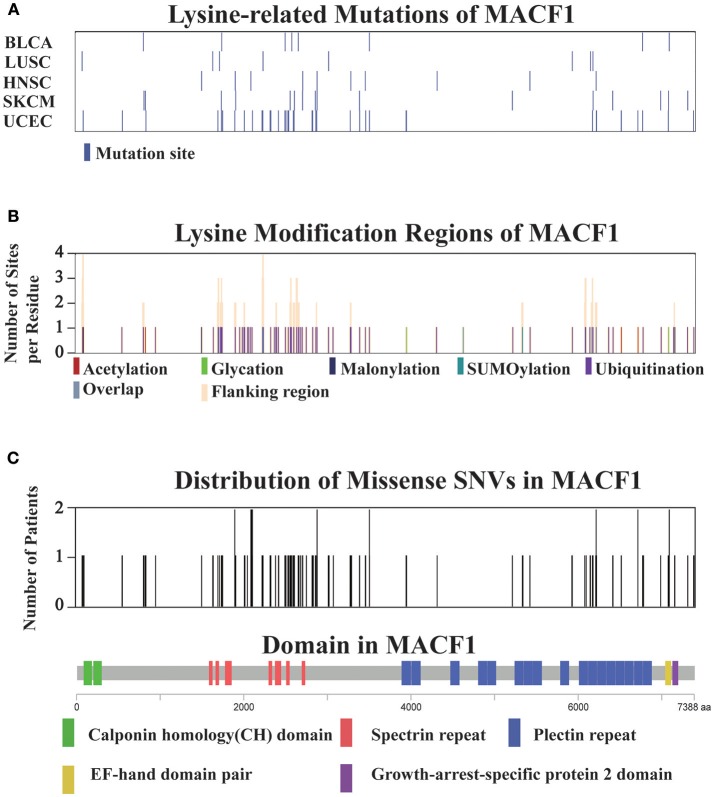
**(A)** Distribution of lysine modification-related mutations in MACF1 across the top five cancer types. **(B)** The lysine modification regions and number of flanking modified sites per residue (orange) in MACF1. **(C)** The number of mutations per residue in MACF1. The domain organization of MACF1 is shown below the chart.

For all 12 selected cancer types, we applied this model to identify potential driver proteins regarding the 7 types of lysine modification. Of the 13,378 mutated proteins, a total of 473 proteins were found to have significantly higher substitution rates in lysine modification motifs than in background regions (Figure 3A). Of these 473 proteins, 45 are known cancer drivers according to the Cancer Gene Census in COSMIC database where there are 699 cancer drivers in total, highlighting that our identified proteins had significant functionality in tumorigenesis (Supplementary Table 4, *p*-value = 1.9 × 10^−5^, Fisher's exact test). Among these driver proteins, 25 were found to be significantly mutated in more than one cancer type (Figure 3B), suggesting a general driver mechanism of lysine modification in multiple cancer types. In our tested cancer types, endometrial carcinoma had the most striking number of lysine modification-related mutations. In total, 86 proteins were identified as significantly mutated in the region of modification motifs across 7 types of lysine modifications (Figure 3A). More than 20 proteins in endometrial carcinoma had a mutation rate in lysine modification motifs that was higher than 2% (Figure 3C). Moreover, we found that in endometrial carcinoma, the most frequently mutated gene, *MACF1*, also had a high lysine modification-related mutation rate in other cancers (Figure 4A), including BLCA, LUSC, HNSC, and SKCM. According to published literature (Karakesisoglou et al., 2000), the coding product of *MACF1* can facilitate actin-microtubule interactions and couple the microtubule network to cellular junctions. Some related works indicated that *MACF1* was an important signaling molecule with various functions in cell processes, embryo development, tissue-specific functions, and human diseases (Hu et al., 2016). Since *MACF1* can act as a positive regulator in the Wnt receptor signaling pathway and function through the oncogenic MAPK signaling pathway (Chen et al., 2006), it has been selected as a novel potential target in several cancers (Miao et al., 2017). In our studies, various types of lysine modifications were mapped to *MACF1*, indicating an important function of post-translational modification in regulating the formation and interaction of cytoskeletal networks (Figure 4B). Interestingly, in our analysis, we observed a remarkable distribution of amino acid mutations around the lysine modification sites across 12 cancer types (Figure 4C). Moreover, most of the lysine modification-related mutations were found to be located in important functional domains, such as plectin repeats and growth-arrest-specific domains (Figure 4C). The above results suggested that lysine modification-related mutations in *MACF1* may interfere with its proper function and cause the appearance of cancer phenotypes.

### Lysine modification-related mutations are highly conserved and highly deleterious

**Figure 5:**
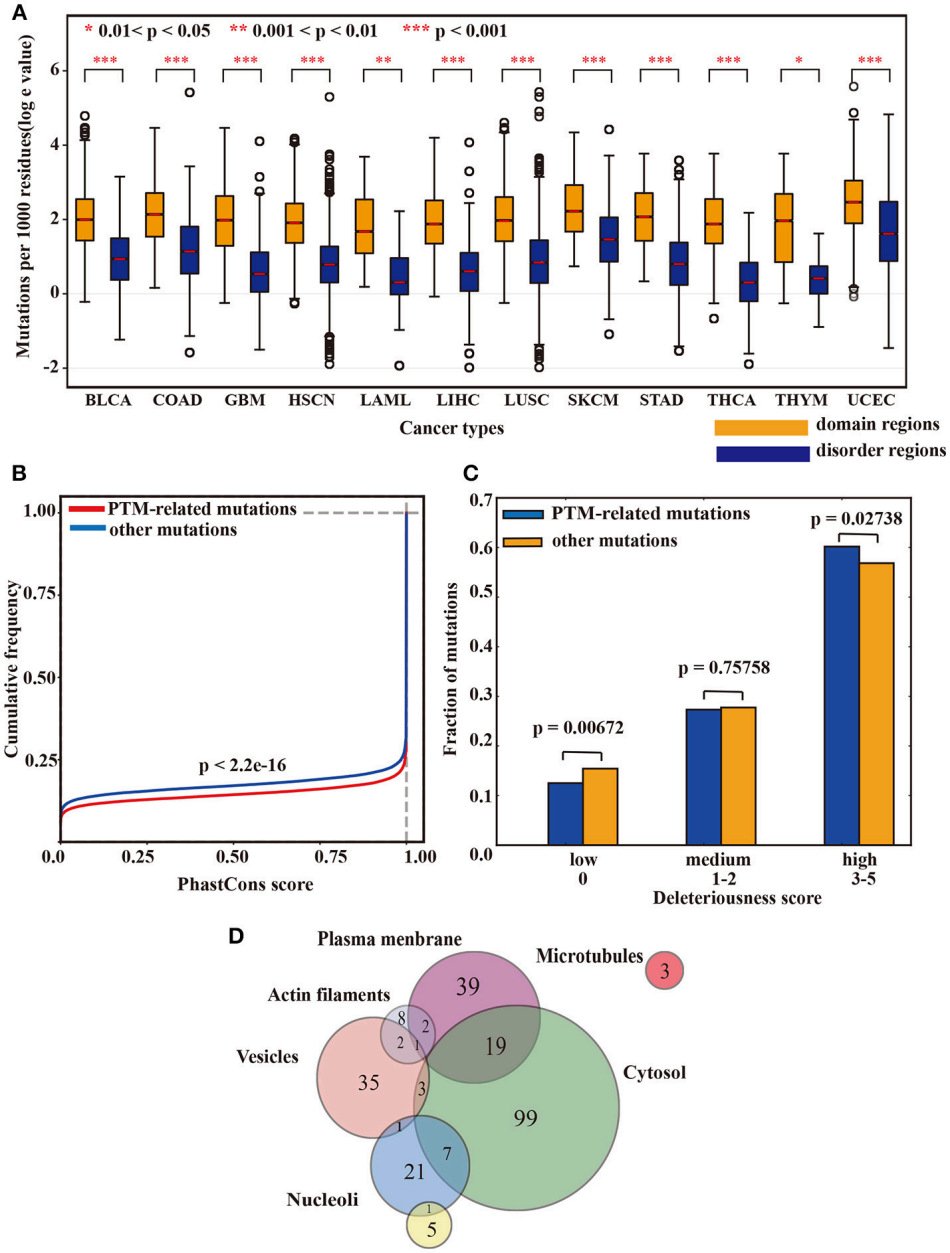
**(A)** The box plot shows the differences in mutation rates in the domain regions and disorder regions. **(B)** The cumulative distribution function of the predicted conservation scores in lysine modification-related mutations and other mutations. A Kolmogorov-Smirnov test was applied to examine their statistical differences. **(C)** The deleteriousness of lysine modification-related mutations and other mutations. A two-tailed population test was applied to evaluate the differences. **(D)** The subcellular localization of the driver proteins that carried a high rate of lysine modification-related mutations.

To explore the potential function of our identified lysine modification-related mutations, we conducted a series of proteome-wide analysis in this study. First, a bootstrap test was applied to examine the functional impact of lysine modification-related mutations on protein domains. Interestingly, among the 12 tested cancer types, lysine modification-related mutations more preferably occurred in known domain regions than in other regions (Figure 5A), suggesting that these kinds of mutations may have underlying effects on protein functions in different cancer types. Furthermore, using hypergeometric test, we also filtered out a set of protein domains that were more concentrated with lysine modification-related mutations. Of which, the Myotonic dystrophy protein kinase domain in *CDC42BPB* (Zhao and Manser, 2015) and AT hook domain in *SETBP1* (Piazza et al., 2013; Coccaro et al., 2017) stand out as two most representative examples (Supplementary Table 5). We expected that this candidate list of mutated domains may help to discover novel mechanisms of abnormal lysine modifications on regulating protein domain functions.

In addition to domain analysis, we also examined the evolutionary conservation of these lysine modification-related mutations. Using the phastCons 100-way scheme (Siepel et al., 2005), we calculated the conservation scores in both lysine modification-related mutations and other non-synonymous mutations and found that mutations related to lysine modification were more functionally conserved than other mutations (*p*-value < 2.2 × 10^−16^, Kolmogorov-Smirnov test) (Figure 5B). Moreover, the deleteriousness level of both lysine modification-related mutations and other ordinary mutations was measured in Figure 5C. As expected, lysine modification-related mutations were predicted to be more deleterious than other ordinary mutations with a two-tailed population test (Figure 5C). Taking together the above three analyses, we can conclude that lysine modification-related mutations may have important roles in regulating many hallmark cancer pathways (Knittle et al., 2017; Chen et al., 2018; Cho et al., 2018) and may be driven by strong positive selection during the development of cancer cachexia.

In addition, to further characterize the cellular function of our identified driver proteins, an analysis of subcellular location was also carried out according to Thul's paper (Thul and Akesson, 2017). For the 473 identified driver proteins, 173 (36.57%) were localized in at least one cell compartment. Among them, 128 (27.06%) were localized in the cytosol, 61 (12.89%) were found in the plasma membrane and 42 (8.87%) were in the vesicles (Figure 5D). Specifically, a majority of our identified proteins (443 out of 473, 93.66%) were outside the nucleus, revealing that identified driver proteins involved in tumorigenesis mostly are non-histone and supporting the idea that abnormal lysine modifications on non-histone proteins also played an indispensable role in cancer development (Singh et al., 2010; Carlson and Gozani, 2016). Further annotation with HistoneDB2.0 revealed that only one protein named H2B1M was histone.

### Pathway analysis reveals underlying roles of lysine modification-related mutations

**Figure 6:**
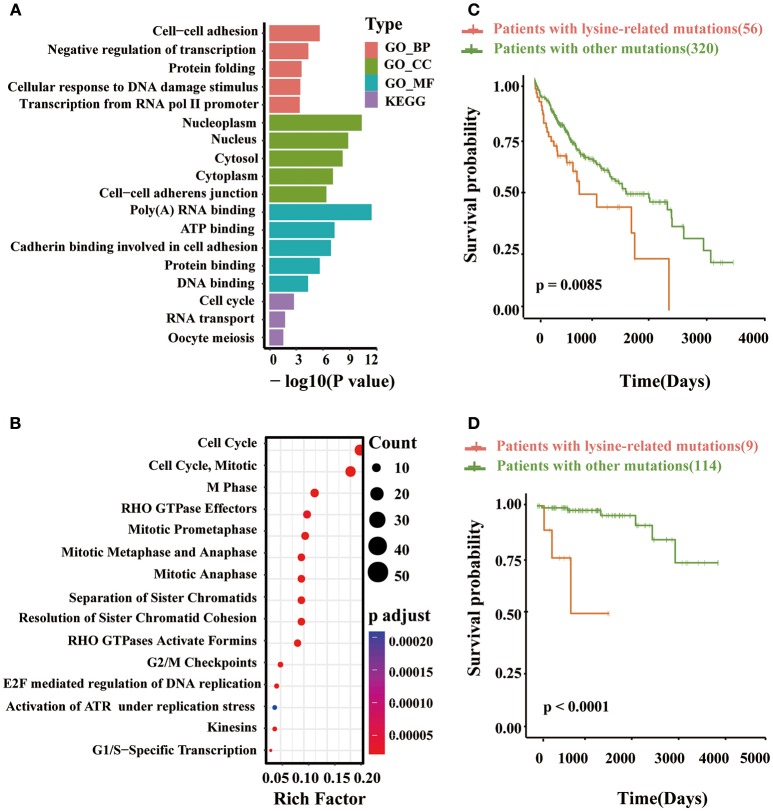
**(A)** The enriched GO terms and KEGG pathways obtained from the identified lysine modification-related driver proteins. **(B)** The result of Reactome pathways analysis on the predicted driver proteins. **(C)** Kaplan–Meier plots comparing the overall survival rates between patients with lysine modification-related mutations and patients without mutations in liver cancer and **(D)** thymic carcinoma.

Based on the identified driver genes, we next preformed pathway analysis to explore network signaling driven by lysine modifications in multiple cancer types. Interestingly, in KEGG annotation, we found that lysine modification-related mutations were significantly enriched in pathways such as cell cycle and RNA transport (Figure 6A). According to published literature (Senderowicz, 2002; van Kouwenhove et al., 2011; Williams and Stoeber, 2012), these pathways were known to have important regulatory roles in the proliferation and apoptosis of cancer tissue. Recently, some driver proteins in these pathway, for example the *MYC* and *EGFR* antagonists, were also being developed as therapeutic agents for prostate and colorectal cancer (Moroni et al., 2005; Vita and Henriksson, 2006; Ciardiello and Tortora, 2008; Perez et al., 2011). Similarly, GO enrichment analysis also indicated that these driver proteins are more likely to participate in cellular processes related to tumorigenesis, including those involved in cell-cell adhesion, negative regulation of transcription, cellular response to DNA damage stimulus and transcription from RNA pol II promoter. As reported in previous studies, the abnormal cell-cell adhesion can serve as one of the 10 special characteristics of cancer and reduced intercellular adhesiveness is indispensable for cancer invasion and metastasis (Hirohashi and Kanai, 2003; Farahani et al., 2014; Lin and Gregory, 2015). Besides, the negative regulation of transcription pathway has been reported to be related with proliferation and apoptosis of cancer cells (Lin and Gregory, 2015; Özeş et al., 2016). Also, the cellular response to DNA damage stimulus is important for maintaining genome stability (Siveen et al., 2014; Lin and Gregory, 2015; Özeş et al., 2016). For the enriched GO term, the transcription from RNA pol II, has been proved as a highly regulated process for tumorigenesis, and can regulate the transcript level of some known oncogenes such as *MYC* (Jonkers and Lis, 2015). A consistent result were also observed in the enrichment analysis of molecular function and cellular component aspects. Our identified driver proteins mainly located in Nucleoplasm, Nucleus Cytosol, Cytoplasm and cell adherens junction, and mainly regulated the RNA-binding or cell adhesion functions. Moreover, Reactome pathway analysis further suggested that mutation of lysine modifications on these proteins can affect cell cycle and mitotic process, especially those associated with cell apoptosis (i.e., the G1/S and G2/M checkpoints) (Bannister and Kouzarides, 2011; Fiandalo and Kyprianou, 2012; Williams and Stoeber, 2012; Figure 6B).

In summary, the above pathway analyses confirmed that proteins with significant lysine modification mutations take part in several critical regulation processes related to cancer phenotypes, such as cell cycle progression and transcript regulation. Particularly, several important driver proteins were found to be dysfunctional in the above cancer-related pathways, which was mainly due to the mutation of specific lysine modification processes as reported by previous experimental studies. Polyubiquitination mutation in PCNA (Cazzalini et al., 2014) and abnormal acetylation in ATM (Bakkenist and Kastan, 2003; Sun et al., 2007) are two representative examples. These results have further confirmed the validity of our computational models.

### Clinical implications of lysine modification dysregulation in cancer patients

Currently, the clinical implications of lysine modifications were largely unknown in multiple cancer types; therefore, here, we performed a systematic investigation to explore the clinical significance of lysine modification processes across 12 cancer types. To perform this investigation, 7,786 clinical data were first collected from TCGA patients. The identified lysine modification-related mutations were then mapped to their corresponding patients according to their recorded barcodes, and the prognosis of both the mutated group and non-mutated group were compared using Kaplan-Meier curves. Of the 12 selected cancer types, we found that patients with lysine modification-related mutations had significantly worse prognosis in liver cancer (LIHC) (Figure 6C) and thymus cancer (THYM) (Figure 6D). The significant correlation between lysine modification-related driver proteins and clinical prognosis observed in these cancer types further indicated the critical implications of lysine modifications in tumorigenesis. Given the above results, we further analyzed the implications of the identified proteins in these two cancer types. Specifically, in liver cancer, we predicted 41 driver proteins that were significantly mutated at lysine modification motifs. Eight (19.5%) of these proteins were reported previously as cancer drivers (Friedenson, 2005; Silvera et al., 2010; Zhou et al., 2011; Chang et al., 2013; Yu et al., 2013; Pandey et al., 2016; Miao et al., 2017; Tang et al., 2017). In particular, *HOXC10* was known to be associated with a decrease in the overall survival rate of liver cancer (Tang et al., 2017). Similar results were also observed in patients with thymus cancer. In total, we identified 8 lysine modification-related driver proteins, and 37.5% (3 out of 8) of them were proven by previous publication (Heyd and Lynch, 2011; Blachly and Baiocchi, 2014; Park et al., 2014; Papoudou-Bai et al., 2016). Again, the *CCAR2* in our identified list is also known to correlate with patient prognosis (Park et al., 2014; Papoudou-Bai et al., 2016). In this regard, we can conclude that our method is sensitive to finding potential gene products that have strong clinical implications in cancer patients. Our computational model may provide useful candidates for cancer diagnosis and therapies.

### Network analysis identified potential downstream targets for our predicted driver proteins

As the lysine modification-related driver proteins may function crucially in several cancer hallmark pathways, it is indispensable to exploit their potential downstream targets through regulation networks in cancer samples. We believed that this step was critical for understanding complex biological networks in cancer patients and could help identify novel drugs and targets for cancer treatments. In this view, we applied a random walk with restart (RWR) algorithm in this section. First, we collected 199,734 pairs of experimentally validated protein-protein interactions from the STRING database (Szklarczyk et al., 2015) and 17,800 pairs of drug-target interactions from the DrugBank database (Law et al., 2013). These interactions were then combined into a heterogeneous network for subsequent RWR search. Starting from our identified driver proteins, RWR searched through the whole network and determined neighboring targets and drugs that were potentially regulated by the inputted proteins (Supplementary Methods). The search results from 7 types of lysine modifications were presented using Cytoscape software. In total, 2,511 pairs of interactions were selected from the original heterogeneous network.

**Figure 7:**
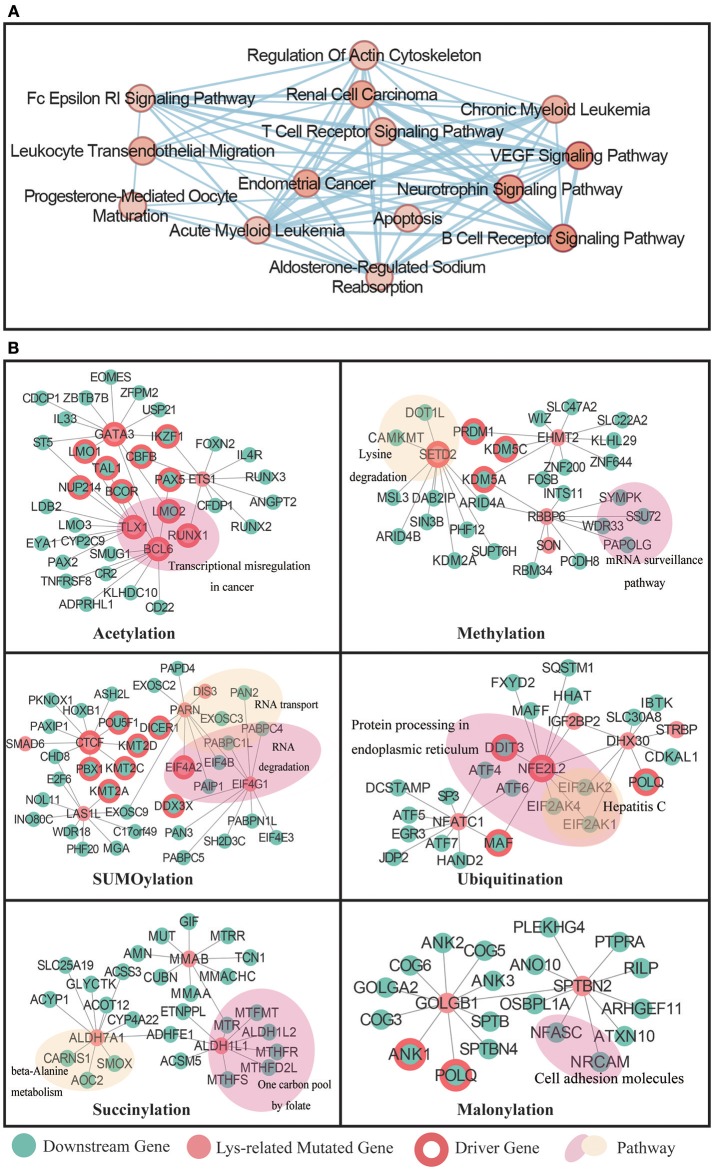
**(A)** Enrichment Map showing the annotated pathways in the whole network. Nodes represent a specific pathway, and edges connect pathways with common genes. **(B)** The RWR analysis result for 7 types of lysine modifications. The identified driver proteins were taken as initial seeds in the RWR process. The predicted targets were labeled in green, the known cancer driver genes were labeled with red circles, and enriched pathways were labeled with a colored shading.

To comprehensively investigate the functional role of the selected network, we applied the Enrichment Map (Zhang et al., 2018) to cluster pathways from the RWR results. Interestingly, 14 pathways were identified as significantly enriched in our lysine modification-mediated network. A majority of these pathways were important cellular processes known to be associated with cancer misregulation (Figure 7A). For example, the VEGF signaling pathway (Hartsough et al., 2013), apoptosis (Lee et al., 2015), and T cell receptor signaling pathway (Friend et al., 2014; Hu and Sun, 2016) are three identified processes that regulate cancer cell proliferation.

Next, to understand network details, we further extracted the subnetwork of each studied modification type by reserving the top 10 most relevant downstream targets (Fouss et al., 2012; Zhu et al., 2013). Specifically, in acetylation, RWR identified 32 downstream targets that may interact with the inputted driver proteins. Among these 32 downstream targets, 12 were well-known cancer drivers. Moreover, 10 of the known cancer drivers were transcription factors related to various cancer types, which agreed with our functional enrichment analysis results that acetylation mutations may affect translational misregulation in cancer (Figure 7B).

Interesting results were also obtained from the analysis of methylation-mutated proteins. Methylation mutations were found to be related to the lysine degradation pathway and mRNA surveillance pathway (Figure 7B). According to published literature (Dimitrova et al., 2015; Shen et al., 2017), lysine demethylases mainly regulate chromatin organization to influence transcriptional processes, and cellular differentiation. Therefore, abnormalities in the lysine degradation pathway may cause serious diseases, such as cancer. In addition, the mRNA surveillance pathway was also reported to be critical in cancer development (Popp and Maquat, 2017). Under normal circumstances, the mRNA surveillance pathway can ensure the quality of transcripts and fine-tune transcript abundance in the process of cell metabolism. However, in some cases, tumors will exploit this pathway to downregulate gene expression by apparently selecting for mutations that cause the destruction of key tumor-suppressor mRNAs.

As for SUMOylation, we identified 9 downstream targets that were known to drive cancer development. Further functional analysis revealed that these downstream targets mainly functioned in pathways that controlled RNA metabolism, such as RNA transport and RNA degradation processes. It is well known that in tumor samples, the malignant phenotype is largely the consequence of dysregulated gene expression (Raza et al., 2015). Of all the molecules that affect gene expression, the dysfunction of *EIF4A2* was already known as a key mechanism that may regulate malignant transformation (Eberle et al., 1997). In addition, because of that knowledge, there is a developing focus on targeting *EIF4A* in cancer therapy. In our computational study, we found that aberrant SUMOylation on *EIF4A2* may contribute to the degradation process of RNA transcripts, representing an interesting candidate for further experimental verification.

For other lysine modifications, such as ubiquitination, succinylation, and malonylation, the RWR method also identified many potential downstream targets that may play crucial roles in cancer-related pathways. For example, in ubiquitination, protein processing in the endoplasmic reticulum was identified as related to ubiquitination mutations in cancer patients. Two critical driver genes were found to be associated with upstream ubiquitination-related mutations. Additionally, succinylation-related mutations were predicted to regulate the downstream one carbon metabolism pathway. As illustrated in published papers (Kalhan, 2013; Baggott and Tamura, 2015; Pirouzpanah et al., 2015), this cancer pathway is not only essential for the de novo synthesis of purines but also significantly related to the expression of driver genes in breast cancer patients. Furthermore, malonylation mutations were shown to influence cell adhesion molecules and components in the Golgi complex (Figure 7B), which may correlate to the metastasis of cancers. In summary, our method identified a series of potential downstream proteins that were expected to correlate to lysine modification mutations. Some of these proteins were identified in previous publications, whereas others may be good candidates for follow-up experimental studies. We hope that a deeper investigation of these candidates will help illuminate novel mechanisms in cancer biology.

In addition to lysine modification-mediated downstream targets, RWR analysis also identified 13 corresponding drugs that were considered to be affected by lysine modification mutations (Supplementary Table 6). Of the 13 identified drugs, 7 were reported to be antineoplastic agents. They are azacitidine (Cortvrindt et al., 1987), acadesine (Montraveta et al., 2014), Cytidine (Periyasamy et al., 2015), mizoribine (Franchetti et al., 2005), titanium dioxide (Tyagi et al., 2016), Cytarabine (Xie et al., 2015), and Zebularine (Sabatino et al., 2013). As a remarkable therapeutic drug, Zebularine can produce an impressive therapeutic effect through the induction of apoptosis in several cancers, such as lymphoma (Montraveta et al., 2014, 2015), leukemia (Robert et al., 2009), retinoblastoma (Theodoropoulou et al., 2013), and colorectal cancer (Ly et al., 2013).

## Discussion

Since mutations in cancer-driven genes perform a crucial promoting effect in the process of cancer development, the prediction of such genes is of great importance in both the theoretical study of complex diseases and the clinical diagnosis of cancer patients (Kelly et al., 2010; Dawson and Kouzarides, 2012; Di Martile et al., 2016). Instead of simply identifying cancer drivers that carried exceeded number of mutations, our studies further considered the corresponding functional consequences of a given mutation in the form of lysine modifications. A hierarchical Bayesian model was therefore established to predict mutations that can alter lysine modification level. Unlike other frequency-based methods, our model did not require to accurately interpret the background mutation rate, which make it more appropriate for identifying rare mutations in the modification motif regions. By using our computational model, we have identified numbers of amino acid mutations that located in the lysine modification motifs. Based on the lysine modification-related mutations, we also identified a set of proteins that may probably drive the development of cancer. The subsequent pathway analysis revealed the functional importance of our identified driver proteins. And the survival analysis also confirmed that these proteins may have clinical implications on cancer patients. When annotating the subcellular location of these proteins, we found that vast majority of them were distributed outside nucleus. This is mainly because that most of the identified drivers were non-histone proteins, and may participate in various cellular processes across cytoplasm. For lysine modified proteins, one of the most special type was histones. In previous literatures (Strahl and Allis, 2000; Tan et al., 2011), a huge catalog of histone modifications have been described, especially lysine modification. In our data sets, 376 lysine modification sites were collected from histones, and 349 of them were found to have lysine modification-related mutations in cancer patients. The remaining 99.8% mutation sites were located in 472 non-histone proteins. This result indicated that the abnormal lysine modification on non-histone proteins may also have critical role on regulating cancer progression. Further experimental verification on these non-histone proteins will assist the discovery of novel mechanisms for the pathogenesis of multiple cancers. Based on the above lysine modification-related mutations and cancer driver proteins, we further explore their downstream targets through a heterogeneous network using the RWR algorithm. As we expected, searching the PPI and drug-target network can help us identify potential treatment agents for cancer therapeutics. For instance, the azacitidine and acadesine. Azacitidine has been study as antiproliferative agent in murine B16 melanoma by effecting several cellular metabolic pathways (Cortvrindt et al., 1987), including the activities of S-adenosylmethionine methyltransferase and orotidine-5′-monophosphate decarboxylase (Cihák, 1974; Christman et al., 1983). Acadesine has shown antitumoral effects in the majority of MCL cell lines and primary MCL samples via modulating immune response, actin cytoskeleton organization and metal binding (Montraveta et al., 2014). We believed that the integration of our computational resources with other downstream analysis methods or experimental studies may contribute to expand the prospects of lysine modification in cancer studies, and may also open new avenues for cancer therapeutics.

Although satisfying results were obtained in our analysis, several considerations still limit the interpretation of our discoveries. First, our current analysis only involved non-synonymous point mutations, which were the simplest to interpret, and removed other mutation types, such as frameshift variations, deletions and insertions. However, these other types of mutations can also induce carcinogenesis, so these mutations must be included in our computational model to comprehensively interpret the functional role of lysine modification processes. Second, as somatic mutations are rare in some proteins, bias will be introduced and false positives will increase in such cases. Expanding the data volumes and covering as many cancer patients as possible are efficient ways to solve this problem. In the near future, more mutation samples will be included in the analysis, and the accuracies of our computational model can be improved. Third, at this stage, our original computational model only evaluated mutations located in modification motifs. As post-translational modification processes are mainly catalyzed by specific enzymatic systems, another valuable factor for interpreting mechanisms in cancer development will be considering upstream mutations that can alter enzyme activities (Li et al., 2010; Prabakaran et al., 2012). Therefore, constructing a complete model that not only identifies substrate mutations but also analyzes enzymatic alterations is a priority task in future studies.

In summary, the above analysis highlights a new tumorigenesis mechanism through the misregulation of lysine modifications in cancer-relevant pathways. We found that mutations at lysine modification sites significantly correlated with worse overall survival in several cancers, indicating that mutated proteins identified by our model can function as novel potential cancer drivers and can be used as diagnostic biomarkers in clinical practice. Overall, we expect that the integration of PTM data and cancer mutations by our proposed method can provide further functional evidence not available from traditional methods to the research community.

## Author contributions

JR and YBX conceived, designed, and supervised all phases of the project. LC, YM, ML, YRZ, ZG, DP, BH, and YYZ performed the bioinformatics analysis. LC and YBX wrote the manuscript. XL, YX, and ZZ contributed to data interpretation, discussions, and editing of the paper. All authors read and approved the final manuscript.

### Conflict of interest statement

The authors declare that the research was conducted in the absence of any commercial or financial relationships that could be construed as a potential conflict of interest.
