# Supplementary material for: Pan-Cancer Analysis Reveals the Functional Importance of Protein Lysine Modification in Cancer Development
Source: Front Genet. 2018 Jul 17;9:254. doi: 10.3389/fgene.2018.00254 (PMC6056651; doi:10.3389/fgene.2018.00254)
Supplement: Supplementary file 7 [file Presentation_1.PDF]

Pan-cancer analysis reveals the functional importance of protein lysine modification in cancer development

Li Chen[1,†], Yanyan Miao [1,†], Mengni Liu[1,†], Yanru Zeng[1], Zijun Gao[1], Di Peng[1], Bosu Hu[1], Xu Li[2], Yueyuan Zheng[1], Yu Xue[3], Zhixiang Zuo[1], Yubin Xie[1,*] and Jian Ren[1,*]

[1]State Key Laboratory of Oncology in South China, Cancer Center, Collaborative Innovation Center for Cancer Medicine, School of Life Sciences, Sun Yat-sen University, Guangzhou 510060, China

[2]Spine Center, Department of Orthopaedics, Anhui Provincial Hospital, the First Affiliated Hospital of USTC, Hefei, Anhui, China

[3]Department of Biomedical Engineering, College of Life Science and Technology, Huazhong University of Science and Technology, Wuhan 430074, China

Running title: Lysine modification-related mutations in cancer

***Correspondence:**

Jian Ren (renjian.sysu@gmail.com)

Yubin Xie (xieyb6@mail.sysu.edu.cn)

[†]These authors have contributed equally to this work.

1 Supplementary Methods

1.1 Determination of the best motif length for 7 types of lysine modifications by k-means algorithm

To cluster the collected lysine modification sites, we first extracted the L-amino acid flanking regions with the modified lysine residue at the center in both positive and negative data set. Specifically, according to our previously published papers (Zhao et al., 2014; Xie et al., 2016), the negative data set was constructed by preserving all lysine residues in the same protein that were not experimentally verified as modified by specific chemical groups. For a given L-amino acid flanking region, there are 23 kinds of possible symbols, including 20 natural amino acids, an unknown amino acid X, a rare amino acid U and a gap character “*”. Accordingly, we can therefore construct a position specific scoring matrix (PSSM) with dimensionality in L×23. Based on these definitions, the following computational processes were performed.

(1) Centroid initialization for K-means

For each modification, all L-amino acid flanking regions were randomly divided into k categories. The number of amino acids *j* observed in the *i*th position for each category was defined as $m_{i,j}$. The conservative score $M_{i,j}$, was calculated as shown in equation 1, and b_j was the background occurrence rate of amino acid *j* obtained from UniProt database (Supplementary Table 1).

$$M_{i,j} = log(\frac{m_{i,j}}{b_j}) \tag{1}$$

For each category, we can build a PSSM of L*23-dimension in equation 2.

$$P_{PSSM} = \begin{bmatrix} M_{1,1} & M_{1,2} & \cdots & M_{1,L} \\ M_{2,1} & M_{2,2} & \cdots & M_{2,L} \\ \vdots & \vdots & \vdots & \vdots \\ M_{23,1} & p_{23,2} & \cdots & M_{23,L} \end{bmatrix} \tag{2}$$

(2) Reclassification of the L-amino acid flanking regions

We re-classified all the L-amino acid flanking regions based on the scheme listed below. Firstly, in each initial category, we calculated the similarity score $R_{peptide}$ of each peptide from the corresponding P_{PSSM} using equation 3. All peptides were then categorized to the corresponding category according to the max score.

$$R_{peptide}=\sum_{i=0}^{L} M_{i,AA} \tag{3}$$

Where $M_{i,AA}$ was the score of a given amino acid at the *i*th position in the corresponding PSSM. Based on the new classification, PSSMs of each cluster were re-calculated using the equations listed in step 1.

.

(3) Iteration step

To obtain optimal clusters, step 2 was executed iteratively when the whole cluster reaching a convergence state. To measure such a convergence state, we defined a euclidean distance as shown in equation 4. The major goal of equation 4 is to quantify the difference of PSSMs between two adjacent iterations.

$$D_{distance}=\frac{\sum_{z=0}^{k}\sum_{i=0}^{L}\sum_{j=0}^{23}(M_{i,j}-N_{i,j})}{2} \tag{4}$$

In the above equation, $M_{i,j}$ was the conservative score of amino acid *j* in the *i*th position in current PSSM, $N_{i,j}$ was the conservative score of amino acid *j* in the *i*th position in previous PSSM. If the difference between two tested PSSMs are less than 1×10^{-8}, and such state continued for more than 50 iterations, we will consider the algorithm converge at the steady status. Otherwise, step 2 will be executed again.

(4) Choose the best clustering result

Since the performance of k-means clustering is very sensitive to initial point selection, we therefore repeated the above steps for several times to obtain a satisfied clustering result. The satisfaction score of each initialization was calculated using equation 5. Of which, *z* is the number of clusters specified in the clustering algorithm. We then kept the clusters with the

highest satisfaction score as the final result.

$$S_{satisfy}=\frac{\sum_{z=0}^{k}\sum_{i=0}^{L}\sum_{j=0}^{23}M_{i,j}}{k}(M_{i,j}>0) \tag{5}$$

(5) Choose the best number of clusters in k-means algorithm

To find the best number of clusters for each modification type, the statistical model proposed by Vacic et al was used (Vacic et al., 2006). In more detail, let P and Q be the positive and negative peptides, respectively. Then, $|P|$ and $|Q|$ were defined as the number of peptides in the corresponding data set. Let P_i denoted the *i*th peptide in the positive dataset P, and $P_{i,j}$ denoted the *j*th position in peptide P_i. For each position in P and for each symbol a from the 23 symbols, a binary vectors $X_p^{j,a}$ can be form by equation 6

$$X_p^{j,a} = \left(I_1, I_2, \cdots, I_{|p|}\right) \tag{6}$$

I_i in equation 6 can be calculated as shown in equation 7.

$$I_i = \begin{cases} 1 & P_{ij} = a \\ 0 & P_{ij} \neq a \end{cases} \tag{7}$$

Vector $X_Q^{j,a}$ was conversely formed. Next, we calculated the p-value under the null hypothesis that vectors $X_p^{j,a}$ and $X_Q^{j,a}$ were sampled from the same distribution using a two sample t-test. Based on the calculated p-value, we can construct the significant PSSM P as shown in equation 8.

$$P_{PSSM} = \begin{bmatrix} p_{1,1} & p_{1,2} & \cdots & p_{1,L} \\ p_{2,1} & p_{2,2} & \cdots & p_{2,L} \\ \vdots & \vdots & \vdots & \vdots \\ p_{23,1} & p_{23,2} & \cdots & p_{23,L} \end{bmatrix} \tag{8}$$

Where L denoted the length of peptides, $p_{i,j}$ was the p-value of the *i*th amino acid in the *j*th position for a given peptide. For each cluster, we obtained a P_{PSSM} with a dimension of 23*L.

Since P_{PSSM} can only represent the differences of sequence conservation between

positive and negative sites, a method that also measured the conservation tendency should be included. Therefore, we further established the following computational processes to address this issue. Firstly, we counted the observed frequency of an amino acid *a* in position *j* for positive and negative data set, respectively. And then, we computed the modified PSSM score using equation 9 and 10.

$$\delta_{i,j} = \frac{f_{i,j}^{Pos} - f_{i,j}^{Neg}}{p_{i,j}} \tag{9}$$

$$E_{i,j} = \begin{cases} ln(\left|\delta_{i,j}\right| + 1) & \delta_{i,j} \geq 0 \\ -ln(\left|\delta_{i,j}\right| + 1) & \delta_{i,j} < 0 \end{cases} \tag{10}$$

Where $f_{i,j}^{Pos}$ and $f_{i,j}^{Neg}$ were the observed frequency of the *i*th symbol in the *j*th position of positive peptides and negative peptides. We next constructed the final modified P_{PSSM} as shown in equation 11.

$$E_{PSSM} = \begin{bmatrix} E_{1,1} & E_{1,2} & \cdots & E_{1,L} \\ E_{2,1} & E_{2,2} & \cdots & E_{2,L} \\ \vdots & \vdots & \vdots & \vdots \\ E_{23,1} & E_{23,2} & \cdots & E_{23,L} \end{bmatrix} \tag{11}$$

In this modified E_{PSSM}, if $E_{i,j}$ is greater than zero, then the corresponding amino acid in the *j*th position is more likely to appear in the positive peptides. While in the opposite case, the amino acid will have a better chance of appearing in the negative peptides. If the absolute value of $E_{i,j}$ is closer to 0, this amino acid was not significantly difference between two datasets.

Finally, we summed up the maximum value of each position in E_{PSSM} to get the conservation score of each cluster as shown in equation 12.

$$score_n = \text{Max}(\sum_{L=0}^{L}(W_{m,n})) \tag{12}$$

Where $score_n$ was the score of the *n*th cluster. We calculated the mean of conservation score for each k-cluster and chose the k-cluster with maximum conservation score as the result

of each modification.

In order to further determine the linear motif length of different types of modification sites, we need to select its optimal upstream and downstream regions. To do this, we plotted the sequence logo by putting the E_{PSSM} of each cluster into Seq2Logo, and selected the best motif length *L* by experience for each cluster.

1.2 Hierarchical Bayesian model for driver protein identification

Using the above computational method, the motif region of each lysine modification type was determined. Overlapped motifs of the same modification type were merged together and regions without any motifs were regarded as modification-free in every protein.

We assumed that mutations on the motif regions would probably damage the lysine modification process, and therefore influence the function of corresponding proteins via PTM-related pathways. Furthermore, if such lysine modification-related mutations are highly correlated with tumor proliferation, they will probably undergo obvious positive selection and unexpectedly high mutation rates will be observed in the motif regions. In view of this, we can identify lysine-modified proteins that drive the cancer development and progression processes by comparing the mutation rates in both motif regions and modification-free regions. To achieve this, a null hypothesis that the mutation rate in the motif region is the same as which in the modification-free region is proposed.

To accurately model the lysine modification-related mutation rates in both regions, we have designed the following statistical model based on a hierarchical Bayesian method. According to the null hypothesis, we need, first of all, to construct the motif region and the modification-free region. Therefore, the sequence of lysine modification motifs and modification-free regions were merged separately and construct a modification region and a background region for each protein (see Figure 2 in the main text).Next, non-synonymous mutations were mapped to both regions, and the number of patients that mutated at each residue for each tested protein were calculated. More formally, let $Y_1, Y_2, \cdots Y_k$ represented the number of patients that mutated on a corresponding position in the modification region of a given protein *p*. Similarly, the mutation

count in the background region can be represented as $Y_{k+1}, Y_{k+2}, \cdots Y_n$ According to this definition, the observed counts *Y* can be described by a Poisson distribution as shown in equation 13 and 14. Where λ_1 and λ_2 were the mutation rates of the modification region and the background region, respectively.

$$Y_i \sim Poisson(\lambda_1) \qquad i=1,2,3,\cdots,k \tag{13}$$

$$Y_i \sim Poisson(\lambda_2) \qquad i=k+1,k+2,\cdots,n \tag{14}$$

As the selective pressure in different sequence regions varies violently, mutation rate can be fluctuated across different positions. To capture these fluctuations, a prior distribution was putted on λ_1 and λ_2 to build a two hierarchical model. Since the Gamma distribution is a conjugate prior for the Poisson distribution, two Gamma distributions with different shape parameter α and scale parameter β were used to describe the distribution of λ_1 and λ_2 in Equation 15 and 16.

$$\lambda_1 \sim Gamma(\alpha_1, \beta_1) \tag{15}$$

$$\lambda_2 \sim Gamma(\alpha_2, \beta_2) \tag{16}$$

A major goal of our model is to obtain estimations of the marginal distribution of λ_1 and λ_2 given the observed data *Y*, *i.e.* calculating $P(\lambda_1|Y)$ and $P(\lambda_2|Y)$. To calculate this, we first need to obtain the full joint distribution of them. According to the Bayesian Theory, the full joint distribution of λ_1 and λ_2 can be constructed as shown in equation 17.

$$\begin{aligned} P(\lambda_1,\lambda_2|Y) &= \frac{P(Y|\lambda_1,\lambda_2)P(\lambda_1,\lambda_2)}{\sum P(Y|\lambda_1,\lambda_2)P(\lambda_1,\lambda_2)} \propto P(Y|\lambda_1,\lambda_2)P(\lambda_1,\lambda_2) \\ &= P(Y_{1:k}|\lambda_1)P(Y_{k:n}|\lambda_2)P(\lambda_1)P(\lambda_2) \end{aligned} \tag{17}$$

Where $P(Y|\lambda_1,\lambda_2)$ was the likelihood of Y. $P(\lambda_1,\lambda_2)$ was the prior distribution of λ_1 and λ_2. We hypothesized that the mutations observed in the modification and background regions were all independent. Accordingly, the full joint distribution can be further simplified as shown in equation 17. As we plugged the probability density function of the Poisson (equation

18) and Gamma (equation 19) distribution into the above equation, concrete from of the full joint probability were given.

$$P(Y) = Possion(\lambda) = e^{-\lambda}\frac{\lambda^{Y}}{Y!} \tag{18}$$

$$P(\lambda) = Gamma(\alpha,\beta) = \frac{\beta^{\alpha}\lambda^{\alpha\ 1}e^{-\beta\lambda}}{\Gamma(\alpha)} \tag{19}$$

Through proper simplification, the full joint probability can be written as shown in equation 20.

$$P(\lambda_1,\lambda_2|Y) = \prod_{i=1}^{k} e^{-\lambda_1}\frac{\lambda_1^{Y_i}}{Y_i!}\prod_{i=k+1}^{n} e^{-\lambda_2}\frac{\lambda_2^{Y_i}}{Y_i!}\frac{\beta_1^{\alpha_1}\lambda_1^{\alpha_1-1}e^{-\beta_1\lambda_1}}{\Gamma(\alpha_1)}\frac{\beta_2^{\alpha_2}\lambda_2^{\alpha_2-1}e^{-\beta_2\lambda_2}}{\Gamma(\alpha_2)} \tag{20}$$

Computing the marginal distribution from the full joint probability required integrating over other unrelated variables in equation 20. Unfortunately, computing integration from equation 20 was generally a formidable analytic problem, and can hardly be done by human hand. A more straightforward way to do so is to use the Markov Chain Monte Carlo (MCMC) method, *i.e.* Gibbs sampling, for estimation (Gelfand et al., 1990; Gelfand and Smith, 1990). Implementation of Gibbs sampling method required the full-conditional probabilities of all variables in equation 20, that is the $P(\lambda_1|Y,\lambda_2)$ and $P(\lambda_1|Y,\lambda_2)$. To obtain these, we should first take the logarithm of both sides in equation 20 to compute them. Equation 21 shows the final computation results.

$$\begin{aligned}
&ln\,P(\lambda_1,\lambda_2|Y) \\
&= \sum_{i=1}^{k}(-\lambda_1 + Y_i\, ln\,\lambda_1 - lnY_i!) + \sum_{i=k+1}^{n}(-\lambda_2 + Y_i\, ln\,\lambda_2 - ln\,Y_i!) + (\alpha_1 - 1)ln\,\lambda_1 \\
&- \beta_1\lambda_1 - ln\Gamma(\alpha_1) + \alpha_1\, ln\,\beta_1 + (\alpha_2 - 1)ln\lambda_2 - \beta_2\lambda_2 - ln\Gamma(\alpha_2) + \alpha_2\, ln\,\beta_2 \\
&= -k\lambda_1 + ln\lambda_1\sum_{i=1}^{k}Y_i - \sum_{i=1}^{k}lnY_i! - (n-k)\lambda_2 + ln\,\lambda_2\sum_{i=k+1}^{n}Y_i - \sum_{i=k+1}^{n}lnY_i! + \\
&+ (\alpha_1 - 1)ln\,\lambda_1 - \beta_1\lambda_1 - ln\Gamma(\alpha_1) + \alpha_1\, ln\,\beta_1 + (\alpha_2 - 1)ln\lambda_2 - \beta_2\lambda_2 - ln\Gamma(\alpha_2) + \alpha_2\, ln\,\beta_2
\end{aligned} \tag{21}$$

As reported in previous publications (George and McCulloch, 1993; Gilks et al., 1996), full conditional probabilities for a given variable can be derived by abstracting out from the joint probability only those elements including that variable and treating other components as constants. Based on this rule, the full conditional posterior probability of λ_1 and λ_2 can be calculated as shown in equation 22 and 23.

$$\begin{aligned} &ln\,P(\lambda_1|\lambda_2,Y) = -k\lambda_1 + ln\,\lambda\sum\nolimits_{i=1}^{k} Y_i + (\alpha_1 - 1)\,ln\,\lambda_1 - \beta_1\lambda_1 \\ &= (\sum\nolimits_{i=1}^{k} Y_i + \alpha_1 - 1)ln\lambda_1 - (k + \beta_1)\lambda_1 \\ &\propto ln\,Gamma(\sum\nolimits_{i=1}^{k} Y_i + \alpha_1, k + \beta_1) \end{aligned} \tag{22}$$

$$\begin{aligned} &ln(\lambda_2|\lambda_1,Y) = -(n-k)\lambda_2 + ln\,\lambda_2\sum\nolimits_{i=k+1}^{n} Y_i + (\alpha_2 - 1)\,ln\,\lambda_2 - \beta_2\lambda_2 \\ &= (\sum\nolimits_{i=k+1}^{n} Y_i + \alpha_2 - 1)\,ln\,\lambda_2 - (n - k + \beta_2)\lambda_2 \\ &\propto ln\,Gamma(\sum\nolimits_{i=k+1}^{n} Y_i + \alpha_2, n - k + \beta_2) \end{aligned} \tag{23}$$

Interestingly, after simplification, the full conditional posterior probabilities of λ_1 and λ_2 are all reduced to gamma distribution from which direct sampling is straightforward (equation 24 and 25).

$$P(\lambda_1 \mid \lambda_2, Y) = Gamma(\sum_{i=1}^{k} Y_i + \alpha_1, k + \beta_1) \tag{24}$$

$$P(\lambda_2 \mid \lambda_1, Y) = Gamma(\sum_{i=k+1}^{n} Y_i + \alpha_2, n - k + \beta_2) \tag{25}$$

To test the difference between the mutation rates of background region and modification region, a variable of interest might be the relative mutation rate defined as $R = \frac{\lambda_1}{\lambda_2}$. Previously, in the published paper from Carlin et al (Carlin et al., 1992), they have given an instruction that if a variable *W* actually appears as a function of another variable *U*, the full conditional probability of *W* can be obtained by univariate transformation from that of *U*. Following these, we further transform λ_1 to R (equation 26) to obtain the full conditional probability of R.

$$\lambda_1 = R\lambda_2 \tag{26}$$

Plugging equation 26 into equation 24, we can obtain equation 27.

$$\begin{aligned} &ln\,P(R|\lambda_1,\lambda_2,Y) = ln\,P(R\lambda_2|\lambda_2,Y) \\ &= -kR\lambda_2 + lnR\lambda_2\sum\nolimits_{i=1}^{k} Y_i + (\alpha_1 - 1)lnR\lambda_2 - \beta_1 R\lambda_2 \\ &= -kR\lambda_2 + (ln\,R + ln\,\lambda_2)\sum\nolimits_{i=1}^{k} Y_i + (\alpha_1 - 1)(ln\,R + ln\,\lambda_2) - \beta_1 R\lambda_2 \end{aligned} \tag{27}$$

Similarly, abstracting R from equation 27, we can reduce the full conditional probability of R to equation 28.

$$
\begin{aligned}
& lnP(R|\lambda_1,\lambda_2,Y) \propto -kR\lambda_2 + ln\,R\sum_{i=1}^{k} Y_i + (\alpha_1 - 1)\ lnR - \beta_1 R\lambda_2 \\
& = (\sum_{i=1}^{k} Y_i + \alpha_1 - 1) ln\,R - (\lambda_2 k + \lambda_2 \beta_1)R \\
& \propto ln\,Gamma(\sum_{i=1}^{k} Y_i + \alpha_1, \lambda_2 k + \lambda_2 \beta_1)
\end{aligned} \tag{28}
$$

Again, the full conditional posterior probability of R is confirmed to gamma distribution (equation 29).

$$
P(R|\lambda_1,\lambda_2,Y) = Gamma(\sum_{i=1}^{k} Y_i + \alpha_1, \lambda_2 k + \lambda_2 \beta_1) \tag{29}
$$

After calculating all the full conditional probabilities of each variable, we can now use Gibbs sampling algorithm, a Markov Chain Monte Carlo (MCMC) method, to sample from Equation 21, 22 and 29 to estimate the marginal distribution of these parameters. The pseudocode is shown in Supplementary Figure 3.

By taking $\alpha_1 = \alpha_2 = 1$ and $\beta_1 = \beta_2 = 0.5$, we started the Gibbs sampling algorithm as shown in Supplementary Figure 3. During the calculation, we totally performed 5,200 iterations and dropped out the first 200 as the burn-in process. Finally, the marginal distribution of λ_1, λ_2 and *R* are estimated by the data sampled from the last 5,000 iterations.

Given the null hypothesis raised at the very beginning of this section, we can rewrite it as shown in equation 30.

$$
\begin{aligned}
& H_0: R \le 1 \\
& H_1: R > 1
\end{aligned} \tag{30}
$$

The p-value under the null hypothesis is then calculated from the marginal distribution of R. For each tested protein, the probability of observing the relative mutation rate less than 1 can be calculated. To control false positive, the Benjamini-Hochberg procedure is applied to each p-value. If the corrected p-value for a given protein is lower than the significant level, *i.e.* 0.05, we will identify it as a significantly mutated protein.

1.3 Preparation of the data set for random walk with restart analysis

To identify potential targets for the lysine modification-related driver proteins, we applied a

random walk with restart (RWR) approach in our study. The drug-target, drug-drug and target-target interactions were incorporated to form a heterogeneous network for analysis. Here, we describe each step of the above procedure in detail.

The drug-target network was compiled from the Drugbank database (Version 5.0.9) (Law et al., 2014) by integrating all the external drug links together. Based on the collected drug-target interactions, we next constructed a drug-target relationship matrix for the subsequent RWR analysis. Each element $A_{i,j}$ in the drug-target relationship matrix represented the interaction status of a given drug-target pair. If drug i is recorded to interact with target j, then the $A_{i,j}$ will be set as 1, otherwise 0. Equation 1 shows an example of the drug-target relationship matrix.

$$DT = \begin{bmatrix} & T_1 & T_2 & T_3 & \cdots & T_m \\ D_1 & 0 & 0 & 0 & \cdots & 0 \\ D_2 & 0 & 1 & 0 & \cdots & 0 \\ D_3 & 1 & 0 & 0 & \cdots & 0 \\ \vdots & 0 & 0 & 1 & \vdots & 1 \\ D_n & 1 & 0 & 0 & \cdots & 1 \end{bmatrix} \tag{31}$$

To construct the network between drugs, we first downloaded all the chemical structures of our collected drugs from the Drugbank database. The Open Babel toolbox (O'Boyle et al., 2011) was then applied to compute the molecular fingerprints for each individual drug structure. The molecular fingerprints encode a molecular structure in a series of binary digits that represent the presence or absence of particular substructures in the molecule. In general, calculations of the molecular fingerprints will allow us to quantitatively determine the structural similarity between two molecules. At present, there are four types of fingerprints available: FP2, FP3, FP4 and MACCS. In this study, the MACCS fingerprint was used. Based on the MACCS fingerprint, we next calculated the similarity between two given drugs using the Tanimoto coefficient (equation 32) (Bajusz et al., 2015).

$$Tanimoto = \frac{A \cdot B}{|A|^2 + |B|^2 - A \cdot B} = \frac{\sum_{i=1}^{k} a_i \times b_i}{\sum_{i=1}^{k} a_i^2 + \sum_{i=1}^{k} b_i^2 - \sum_{i=1}^{k} a_i \times b_i} \quad (32)$$

In the above equation, *A* and *B* represent the binary MACCS fingerprints for drug a and b, respectively. For a given pair of binary variables, the Tanimoto coefficient will be calculated as ranging from 0 to +1, and the value of +1 represents the highest similarity. Similar to the drug-target interactions, the network between drugs can be defined as the drug-drug relationship matrix shown in equation 33. Specifically, element $A_{i,j}$ in this matrix is defined as the Tanimoto similarity between drug *i* and drug *j*. Note that, in this study, the network is required to have no multiple edges or self-edges. Therefore, the diagonal of matrix DD will be directly set to 0.

$$DD = \begin{bmatrix} & D_1 & D_2 & D_3 & \cdots & D_n \\ D_1 & 0 & A_{1,2} & A_{1,3} & \cdots & A_{1,n} \\ D_2 & A_{2,1} & 0 & A_{2,3} & \cdots & A_{2,n} \\ D_3 & A_{3,1} & A_{3,2} & 0 & \cdots & A_{3,n} \\ \vdots & \vdots & \vdots & \vdots & \vdots & \vdots \\ D_n & A_{n,1} & A_{n,2} & A_{n,3} & \cdots & 0 \end{bmatrix} \quad (33)$$

In addition to the drug-drug and drug-target interactions, we also measure the target-target relationship for the RWR process. To expand the searching area, we further integrated the protein-protein interaction data from the STRING database (Szklarczyk et al., 2015; Szklarczyk et al., 2017) in our study. Particularly, to ensure the data quality, only the interactions with confidence scores larger than 0.7 were preserved. Again, according to the recorded interactions, we constructed the relationship matrix as shown in equation 34. Similarly, to avoid self-connection in the target-target network, we let the diagonal of matrix TT be 0.

$$TT = \begin{bmatrix} & T_1 & T_2 & T_3 & \cdots & T_m \\ T_1 & 0 & A_{1,2} & A_{1,3} & \cdots & A_{1,m} \\ T_2 & A_{2,1} & 0 & A_{2,3} & \cdots & A_{2,m} \\ T_3 & A_{3,1} & A_{3,2} & 0 & \cdots & A_{3,m} \\ \vdots & \vdots & \vdots & \vdots & \vdots & \vdots \\ T_m & A_{m,1} & A_{m,2} & A_{m,3} & \cdots & 0 \end{bmatrix} \quad (34)$$

The element $A_{i,j}$ in equation 34 represents the similarity score between two target proteins T_1 and T_2 . We calculated this similarity score using the equation proposed by Bleakley and Yamanishi (Bleakley and Yamanishi, 2009)(equation 35).

$$target\_similarity = \frac{SW(T_1, T_2)}{\sqrt{SW(T_1, T_2)}\sqrt{SW(T_1, T_2)}} \quad (35)$$

Here, $SW(T_1, T_2)$ denotes the Smith-Waterman similarity score between the target protein T_1 and T_2.

1.4 Algorithmic details of the random walk with restart process

The previously constructed target-drug, target-target and drug-drug networks were first combined into an undirected heterogeneous network, and the RWR process was then applied to predict potential downstream targets and drugs related to lysine modification mutations. The RWR simulates a random walker from a set of seed nodes and moves it to its neighbors randomly at each step. After a few round of iterations, the random walker will reach a steady state and provide a probability of reaching that point for each node.

To run the RWR process, we first need to construct the transition matrix M according to Seal's method (Seal et al., 2015). Given the heterogeneous network, we can write the transition matrix as shown in equation 36:

$$M = \begin{bmatrix} W_{TT} & W_{TD} \\ W_{DT} & W_{DD} \end{bmatrix} \quad (36)$$

Where W_{TT} , W_{TD} and W_{DD} are the relationship matrix of the target-target, target-drug and drug-drug networks, respectively. In particular, is the relationship matrix of the drug-target network and is equivalent to the transpose of W_{DT}. Based on the transition matrix M , the RWR process can be formally described as follows:

$$p^{t+1} = (1 - \lambda)Mp^{t} + \lambda p^{0} \quad (37)$$

Where p^t is a vector with an *i*th element representing the probability of finding the walker in node *i* at step *t* and p^0 is the initial probability vector. If there are *k* initial nodes in which the walker will start, we will define these *k* initial nodes as having probabilities of *1/k*, and the remaining nodes will have probabilities of 0 (equation 38). Finally, *λ* is a fixed parameter denoting the restart probability at each iteration step.

$$p^{0} = \left[p_1^0, p_2^0, p_3^0, \cdots, p_n^0\right] = \begin{cases} 1/k & initial \\ 0 & otherwise \end{cases} \quad (38)$$

Then, according to equation 37, p^t is updated iteratively until the difference between p^t and p^{t+1} is less than 10^{-6}. After the RWR iteration, all nodes are sorted by their assigned probabilities, and the top 10 most accessible nodes are maintained as candidates that are interacted with our identified driver proteins (Zhu et al., 2013).

References

Bajusz, D., Rácz, A., and Héberger, K. (2015). Why is Tanimoto index an appropriate choice for fingerprint-based similarity calculations? *Journal of cheminformatics* 7(1), 20.

Bleakley, K., and Yamanishi, Y. (2009). Supervised prediction of drug–target interactions using bipartite local models. *Bioinformatics* 25(18), 2397-2403.

Carlin, B.P., Gelfand, A.E., and Smith, A.F.M. (1992). Hierarchical Bayesian analysis of changepoint problems. *Applied statistics*, 389-405.

Gelfand, A.E., Hills, S.E., Racine-Poon, A., and Smith, A.F.M. (1990). Illustration of Bayesian inference in normal data models using Gibbs sampling. *Journal of the American Statistical Association* 85(412), 972-985.

Gelfand, A.E., and Smith, A.F.M. (1990). Sampling-based approaches to calculating marginal densities. *Journal of the American statistical association* 85(410), 398-409.

George, E.I., and McCulloch, R.E. (1993). Variable selection via Gibbs sampling. *Journal of the American Statistical Association* 88(423), 881-889.

Gilks, W.R., Richardson, S., and Spiegelhalter, D.J. (1996). Introducing markov chain monte carlo. *Markov chain Monte Carlo in practice* 1, 19.

Law, V., Knox, C., Djoumbou, Y., Jewison, T., Guo, A.C., Liu, Y., et al. (2014). DrugBank 4.0: shedding new light on drug metabolism. *Nucleic Acids Res* 42(Database issue), D1091-1097. doi: 10.1093/nar/gkt1068.

O'Boyle, N.M., Banck, M., James, C.A., Morley, C., Vandermeersch, T., and Hutchison, G.R. (2011). Open Babel: An open chemical toolbox. *Journal of cheminformatics* 3(1), 33.

Seal, A., Ahn, Y.-Y., and Wild, D.J. (2015). Optimizing drug–target interaction prediction based on random walk on heterogeneous networks. *Journal of cheminformatics* 7(1), 40.

Szklarczyk, D., Franceschini, A., Wyder, S., Forslund, K., Heller, D., Huerta-Cepas, J., et al. (2015). STRING v10: protein–protein interaction networks, integrated over the tree of life. *Nucleic Acids Research* 43(Database issue), D447-D452. doi: 10.1093/nar/gku1003.

Szklarczyk, D., Morris, J.H., Cook, H., Kuhn, M., Wyder, S., Simonovic, M., et al. (2017). The STRING database in 2017: quality-controlled protein-protein association networks, made broadly accessible. *Nucleic Acids Res* 45(D1), D362-D368. doi:

10.1093/nar/gkw937.

Vacic, V., Iakoucheva, L.M., and Radivojac, P. (2006). Two Sample Logo: a graphical representation of the differences between two sets of sequence alignments. *Bioinformatics* 22(12), 1536-1537.

Xie, Y., Zheng, Y., Li, H., Luo, X., He, Z., Cao, S., et al. (2016). GPS-Lipid: a robust tool for the prediction of multiple lipid modification sites. *Sci Rep* 6, 28249.

Zhao, Q., Xie, Y., Zheng, Y., Jiang, S., Liu, W., Mu, W., et al. (2014). GPS-SUMO: a tool for the prediction of sumoylation sites and SUMO-interaction motifs. *Nucleic Acids Research* 42(Web Server issue), W325.

Zhu, J., Qin, Y., Liu, T., Wang, J., and Zheng, X. (2013). Prioritization of candidate disease genes by topological similarity between disease and protein diffusion profiles. *BMC Bioinformatics* 14(Suppl 5), S5-S5. doi: 10.1186/1471-2105-14-S5-S5.

2 Supplementary Figures

Supplementary Figure 1. The normalized conservation score of each k-cluster for 7 types modification.

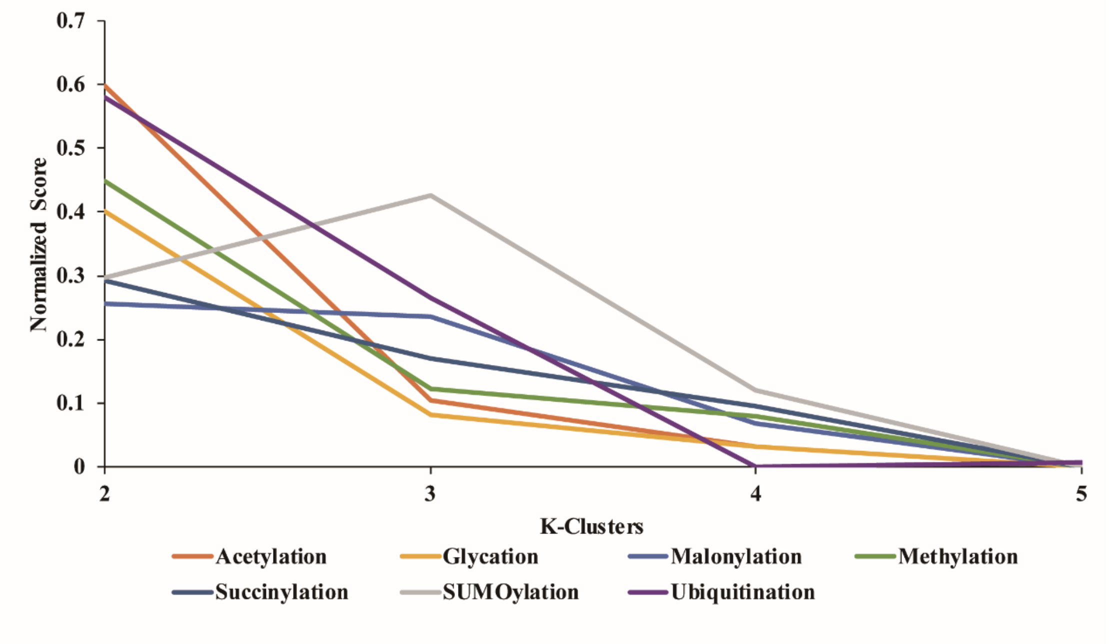

Supplementary Figure 2. The best motifs clustered by k-means for Acetylation, Glycation, Malonylation, Methylation, Succinylation, Ubiquitination and SUMOylation.

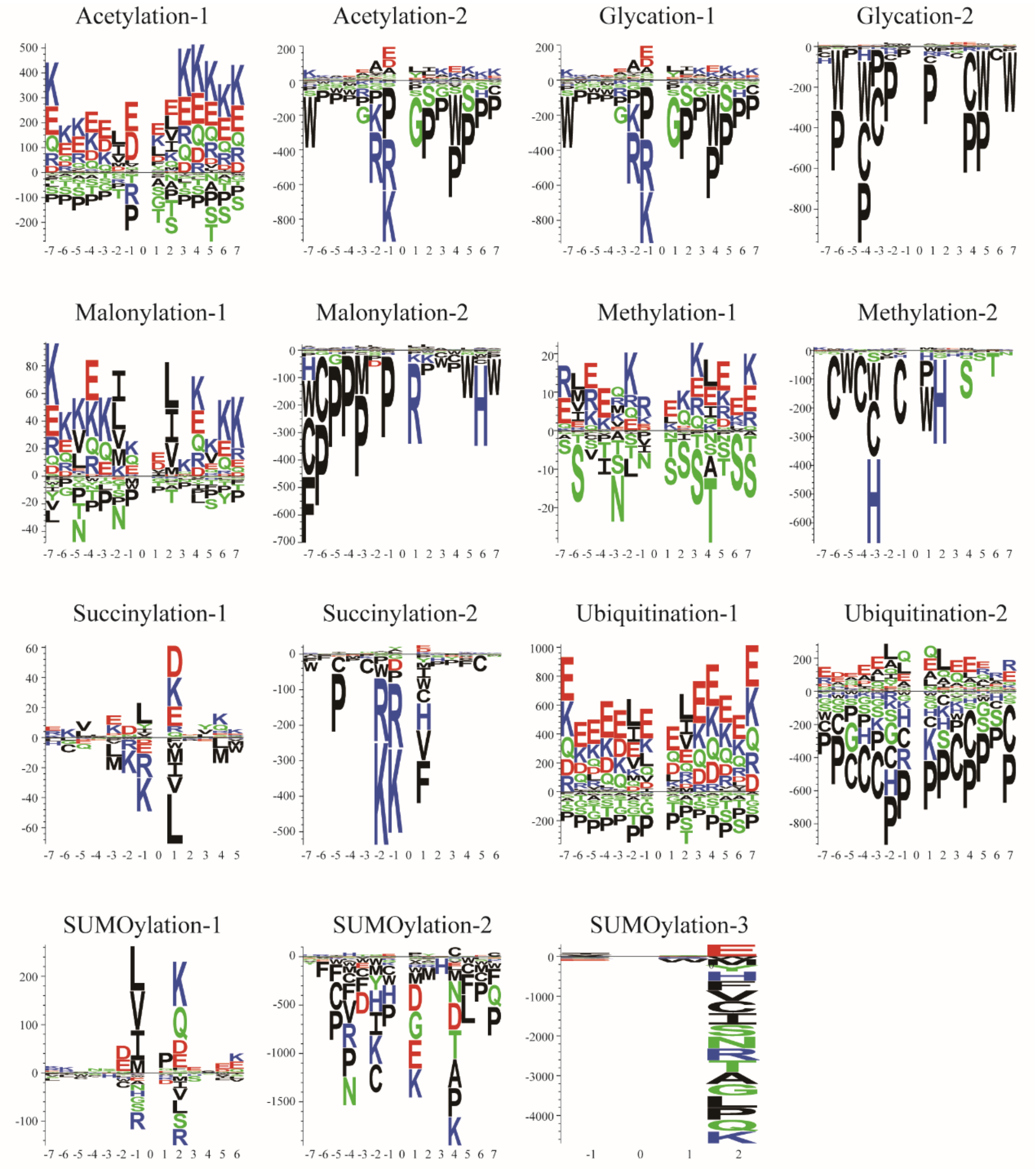

Supplementary Figure 3. The pseudocode of Gibbs sampling in hierarchical Bayesian model

Gibbs sampling

Initialize λ_1, λ_2, R

For iteration i=1, 2, 3, … **do**

$$\lambda_1^{(i)} \sim Gamma(a + \sum_{i=1}^{k} Y_i\ , k + b)$$

$$\lambda_2^{(i)} \sim Gamma(a + \sum_{i=k+1}^{n} Y_i\ , n - k + b)$$

$$R^{(i)} \sim Gamma(a + \sum_{i=1}^{k^{(i)}} Y_i\ , {\lambda_2}^{(i)}(k + \beta_1))$$

End for

3 Supplementary Tables

Supplementary Table 1 – The background rate of 22 amino acids and a gap character used in k-means algorithm.

Supplementary Table 2 – Lysine modification sites collected in this paper.

Supplementary Table 3 – The identified lysine modification-related mutations.

Supplementary Table 4 – The significant lysine modification-related mutated proteins identified using hierarchical Bayesian models.

Supplementary Table 5 – The significantly altered domains analyzed by hypergeometric test

Supplementary Table 6 – The information of drugs explored in network analysis.
